# A Short-Chain Analogue of Seminolipid: Synthesis and Inhibitory Effect on Mouse Fertilization

**DOI:** 10.3390/ph18050611

**Published:** 2025-04-23

**Authors:** Seung Gee Lee, Leila Vahdati, Laura Morelli, Luigi Panza, Federica Compostella, Nongnuj Tanphaichitr

**Affiliations:** 1 Inflammation and Chronic Disease Program, Ottawa Hospital Research Institute, Ottawa, ON K1H8L6, Canada; selee@ohri.ca; 2 Department of Medical Biotechnology and Translational Medicine, University of Milan, Via Saldini 50, 20133 Milan, Italy; l.vahdati@gmail.com (L.V.); laura.morelli@unimi.it (L.M.); 3 Dipartimento di Scienze del Farmaco, Università degli Studi del Piemonte Orientale A. Avogadro, L.go Donegani 2/3, 28100 Novara, Italy; luigi.panza@uniupo.it; 4 Department of Obstetrics & Gynecology, Faculty of Medicine, University of Ottawa, Ottawa, ON K1H8L6, Canada; 5 Department of Biochemistry, Microbiology, Immunology, Faculty of Medicine, University of Ottawa, Ottawa, ON K1H8M5, Canada

**Keywords:** seminolipid, sulfogalactosylglycerolipid, short-chain sulfogalactosylglycerolipid, glycolipids, carbohydrate chemistry, sperm, egg, sperm–zona pellucida binding, fertilization, non-hormonal contraceptive

## Abstract

**Background/Objectives:** Seminolipid (sulfogalactosylglycerolipid (SGG)) is abundantly present on the sperm surface and its roles in sperm–egg interaction are well-documented. SGG liposomes have direct affinity for the zona pellucida (ZP), the egg extracellular matrix. SGG is also integral to the formation of sperm lipid rafts, which are platforms on the sperm surface for ZP binding. Our objective was to chemically synthesize a short-chain analog of SGG (SC-SGG with a C6 acyl chain instead of C16 in the natural lipid), which is solubilized in an aqueous environment, and to determine the inhibitory effects of SC-SGG in mouse sperm–egg interaction, and thus fertilization. **Methods:** SC-SGG was synthesized from a 3-*O*-galactopyranosyl-*sn*-glycerol intermediate protected on the sugar moiety through the acylation of glycerol with caproic acid, deprotection and regioselective 3-*O*-sulfation of the galactose residue. SC-SGG solubilized in a medium was used to treat sperm–egg co-incubates or to pretreat sperm or eggs before co-incubating sperm with eggs or vice versa. Sperm–ZP binding and fertilization (scoring eggs with two pronuclei) were microscopically assessed. **Results:** SC-SGG was efficiently synthesized with a 78% overall yield. SC-SGG inhibited sperm–ZP binding and fertilization of mouse gametes in a concentration-dependent manner, and at 6 µM SC-SGG, the mouse fertilization was zero. SC-SGG inhibited the fertilizing ability of both sperm and eggs, as shown in the pretreatment experiments. **Conclusions:** SC-SGG was an effective inhibitor of mouse fertilization in vitro. It warrants development to be a non-hormonal contraceptive.

## 1. Introduction

Fertilization is the process whereby a spermatozoon and an egg unite to procreate life. Eggs are ovulated as complexes with surrounding cumulus cells interconnected with each other by long-chain glycosaminoglycans, such as hyaluronic acids. Upon entering the female reproductive tract, ejaculated sperm become capacitated and acquire hyperactivated motility patterns. Together with hyaluronidase on the sperm surface to breakdown the long-chain hyaluronic acids, hyperactivated motile sperm can effectively move through the cumulus cell layers to interact with the egg. This interaction, which leads to fertilization, involves a number of steps, starting with recognition between capacitated sperm and the zona pellucida (ZP), the extracellular matrix of mature eggs, in a species-specific manner. Sperm bound to the ZP then traverse through the ZP matrix to enter the egg vitelline space, and a few sperm, all acrosome-reacted, bind to the egg plasma membrane. Then, the plasma membrane of one acrosome-reacted sperm fuse with the egg plasma membrane and the sperm becomes incorporated into the egg proper. This sperm head then forms a male pronucleus inside the egg, whereas the egg metaphase II chromosome becomes the female pronucleus. The formation of the two pronuclei indicates that the egg has been fertilized [1,2,3].

**Figure 1:**
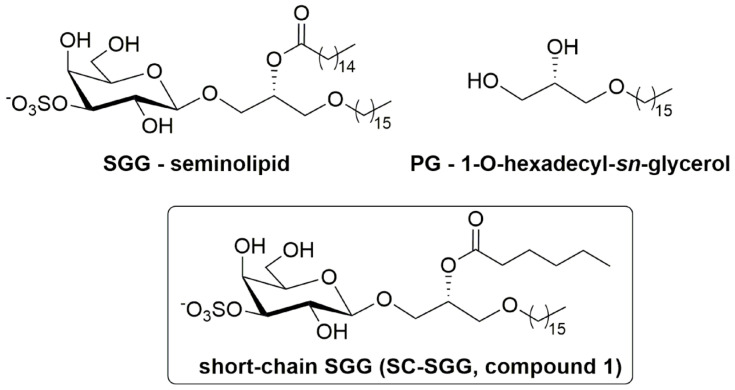
Chemical structure of sulfogalactosylglycerolipid (SGG, seminolipid), short-chain SGG (SC-SGG), and palmitylglycerol (PG). SGG with the structure shown here is the prevalent form in nature, i.e., having a C16:0 acyl chain, whereas our chemically synthesized SC-SGG has an acyl chain of C6:0.

The initial step of sperm–ZP binding has been well-studied. The mammalian ZP consists of only a few glycoproteins, which act as receptors of the binding ligands on the sperm surface [4]. A number of sperm surface and acrosomal molecules, such as SED1 and zonadhesin, have been shown for direct affinity for the ZP [5,6,7,8]. All, except for one, of these molecules are proteins. Uniquely, the molecule of this exception is a sulfoglycolipid, sulfogalactosylglycerolipid (SGG, seminolipid), which exists very selectively on the surface of mammalian testicular germ cells and the head of mature sperm [7,9]. SGG (chemical name: 1-*O*-alkyl-2-*O*-acyl-3-*O*-[3-*O*-oxysulfonyl-β-d-galactopyranosyl]-*sn*-glycerol, Figure 1) has a glycerol backbone with an ether and ester linkage in the *sn*-1 and *sn*-2 position, respectively. The galactosyl-3′-sulfate head group occupies the *sn*-3 position. The alkyl and acyl chains of SGG are predominantly C16:0 (palmityl and palmitoyl group, respectively). The level of SGG in each sperm is about 10 mole% of total sperm lipids, and since SGG exists only in the sperm head, its density in this sperm area is much higher [9]. The binding of sperm to the egg ZP is exclusively in the sperm head area [1], and as a glycolipid, which is generally involved in cell adhesion, we have hypothesized and demonstrated that SGG is involved in sperm–ZP interaction in both mice and humans [10,11]. We have shown that fluorescently labeled SGG liposomes bind directly to the ZP, and SGG liposomes, when co-incubated with sperm and eggs, inhibited sperm–ZP binding in a concentration-dependent manner. Furthermore, sperm incubated with anti-SGG antibodies have a reduced ability to bind to the ZP [10,11].

As an ordered lipid (having saturated hydrocarbon chains as its alkyl and acyl chains), SGG has a propensity to interact with cholesterol and saturated lipids [12], and therefore it is integral in the formation of sperm head lipid rafts, which we have isolated as detergent-resistant membranes (DRMs) or nitrogen-cavitated anterior head plasma membranes (APMs) [12,13,14]. Both DRMs and APMs house a number of ZP-binding proteins together with SGG, and therefore they are sperm head surface platforms that bind to the ZP [7,12,14]. In addition to its role in ZP binding and structural role in sperm lipid raft formation, SGG is involved in recruiting ZP binding proteins into these sperm head surface platforms, either directly or indirectly. In the first case, arylsulfatase a (ARSA, EC 3.1.6.8), a ZP binding protein [15,16,17], can bind SGG with a high affinity (*K*_d_~9 nM) [18], and this is how ARSA in the epididymal lumen fluid deposits onto epididymal sperm (already enriched in SGG on their head surface) during their residence in this organ [19]. Although ARSA is known for its desulfation activity of SGG, this can happen only after SGG is solubilized by a chemical detergent or saposin B (a physiological detergent) [9,20]. Without saposin B present in the epididymal fluid, ARSA presumes to bind to SGG in a non-enzymatic pocket [17]. ARSA and SGG, co-existing in the sperm lipid rafts [12,21], likely act synergistically in ZP binding [6,12]. However, since the molecular ratio of ARSA to SGG is 1:400 [18], the majority of SGG molecules are still either unbound or bound to other ZP proteins. In addition to ARSA, another ZP-binding protein, ACRBP (acrosin-binding protein, sp32) [22], has also demonstrated its direct affinity for SGG [23], and ACRBP is present in the isolated APM vesicles [14]. Like ARSA, ACRBP can act synergistically with SGG in sperm–ZP binding.

In addition to its involvement in sperm–ZP interaction, SGG likely participates in sperm–egg plasma membrane interaction. SGG remains on the post-acrosomal plasma membrane on acrosome-reacted sperm, the site that interacts with the egg plasma membrane [17]. On the egg plasma membrane, there exists SLIP1 (sulfolipid-immobilizing protein 1) [24], the identity of which is determined to be ARSA [15]. The high affinity of ARSA for SGG [18] would be one of the bases of how sperm and egg plasma membranes interact with each other.

Due to its abundance and multiple roles in the formation and functionality of sperm lipid rafts/APMs with affinity for the ZP, SGG, if present exogenously to the sperm and egg suspension, should act as a competitive inhibitor to sperm binding to the ZP. Likewise, exogenous SGG would competitively inhibit sperm–egg plasma membrane interaction. However, natural SGG with two long hydrocarbon chains is hydrophobic and partitions from the aqueous environment to form liposomes of various sizes, making the assessment process of their inhibitory effects on sperm–egg interaction difficult. Furthermore, the administration of SGG liposomes in vivo for this inhibitory purpose is not practical, since the liposomes will likely bind adventitiously to the cell surface they are passing. Therefore, we proposed to chemically synthesize SGG with one chain shortened (e.g., acyl chain), as this short-chain SGG (SC-SGG, compound **1**, Figure 1) will be more homogenously dispersed in an aqueous solution. In this report, we described the chemical synthesis of SC-SGG with C6 acyl chain. This length of acyl chain should still allow the head group (galactosyl sulfate) to be in the same conformation as that of natural SGG [25]. Notably, the chemical synthesis procedures of SGG [26,27,28] or SC-SGG (this report) are less laborious than the extraction and purification procedure of natural SGG from animal tissues [29]. The first objective of our study was to investigate whether SC-SGG could competitively inhibit sperm–ZP binding, and thus fertilization in vitro.

In addition to the putative inhibitory effects on sperm–egg interaction, SC-SGG may affect the membrane structural integrity of the sperm head surface, where SGG is localized. This postulation is based on the previous observation that a fluorescently labeled sulfogalactosylceramide (SGC) can be incorporated into the sperm head plasma membrane possibly through its interaction with SGG [30]. SC-SGG may have a similar activity, and this would affect the structural integrity of the sperm head surface and consequently sperm physiology. Therefore, the effect of SC-SGG treatment of sperm was also investigated in this report.

## 2. Results

### 2.1. Design and Synthesis of SC-SGG

The study of sperm–egg intercellular recognition mediated by SGG is in part hindered by its insolubility in water. For this reason, and to avoid the use of SGG liposomes as outlined in the Introduction, we have designed a short-chain SGG (SC-SGG, compound **1**, Figure 1) as a more aqueous soluble mimic of the natural compound. compound **1** maintains the 3-sulfo-β-d-galactoside carbohydrate head group linked to 1-hexadecyl-*sn*-glycerol, and differs from the natural compound only for the length of the acyl chain, C6 instead of C16. The selection of the C6 acyl chain for SC-SGG came from our logP calculation, used to predict lipophilicity of a lipid with various acyl chain length. As expected, SGG with a natural C16 acyl chain had a logP value twofold higher than that of SC-SGG with a C6 acyl chain (8.38 versus 3.93). This implicated increased solubility of SC-SGG in an aqueous environment. However, shortening the acyl chain of SGG to be C4 did not significantly decrease the logP value (3.79). A similar result was obtained from the calculation of logD (as a measure of lipophilicity of an ionizable compound at the reference pH of 7.4) (see Supplementary Material Part 3). Therefore, SC-SGG was synthesized with the C6 acyl chain. The C6 acyl chain in SC-SGG may also function better than the C4 acyl chain in maintaining the conformation of the head group of SC-SGG to be similar to that of the natural SGG (C16) molecular species.

**Figure 2:**
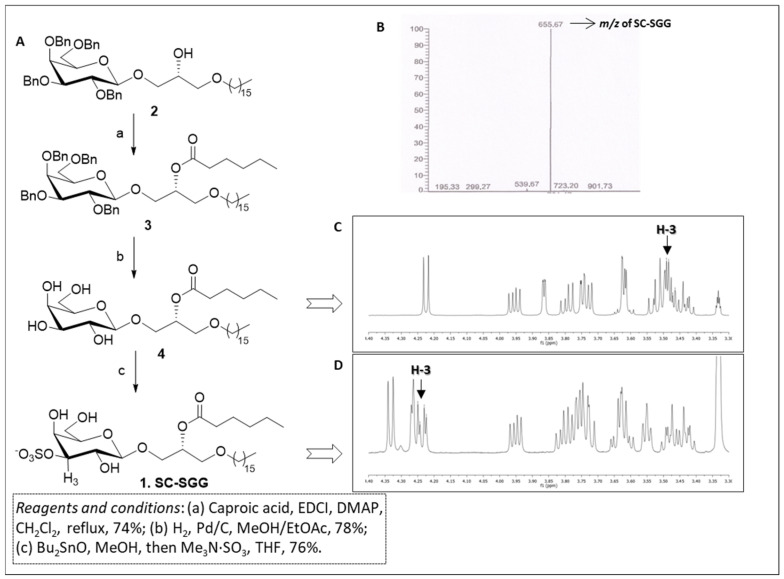
(**A**) Synthetic scheme for compound **1** (SC-SGG). (**B**) Electrospray ionization–mass spectrometry (ESI-MS) spectra (in the negative ion mode) of SC-SGG, revealing the expected *m*/*z* of 655.67. (**C**) ^1^H-NMR spectrum of compound **4**. (**D**) ^1^H-NMR spectrum of SC-SGG showing the de-shielding effect on H-3, due to the introduction of the electron-withdrawing sulfate in position 3 of galactose (compound **1**, H-3 is the proton in position 3 of galactose).

The synthesis of short-chain SGG **1** was carried out through a short and efficient synthetic scheme outlined in Figure 2A. Our synthesis started from the known galactolipid, compound **2**, which was a useful precursor of our target compound, and was obtained following a synthetic procedure described by Konradsson et al. [31]. Compound **2** was characterized by the β-galactosylglycerol backbone of the target SC-SGG with the C16 (palmityl) ether chain present in the scaffold. In addition, the hydroxyl groups of the galactose moiety were protected as benzyl ethers, while the secondary hydroxyl group of glycerol was free for derivatization. Compound **2** was acylated with caproic acid in the presence of soluble carbodiimide and dimethylaminopyridine to efficiently yield the protected galactoglycerolipid **3**. The introduction of the ester moiety is clearly demonstrated by the presence of the signal at 2.35–2.23 ppm in the ^1^H-NMR spectrum due to the CH_2_ group adjacent to the carbonyl function of the ester and by the signal of the carbonyl carbon at 173.2 ppm in the ^13^C-NMR. Moreover, the proton on the central carbon of the glycerol part (H-b) where the ester was attached was shifted to 5.24–5.15 ppm in the ^1^H-NMR spectrum because of the electron withdrawing effect of the ester function. Compound **3** was then subjected to hydrogenolysis to remove the benzyl-protecting groups on galactose, thus producing compound **4**. The disappearance of the signals belonging to the benzyl groups both in ^1^H- and in ^13^C-NMR, together with the retention of the other signals described above for compound **3**, confirmed the structure of compound **4**. Finally, the sulfate group at the 3′ position of galactose ring was introduced by activation of the sugar hydroxyls in the 3′ and 4′ positions with dibutyltin oxide followed by treatment of the dibutylstannylene acetal with the trimethylamine sulfur trioxide-sulfating complex (Me_3_N·SO_3_) [32]. The reaction yielded SC-SGG (**1**) with a 76% yield, which was recovered as a pure compound after purification through flash column chromatography. The purity and identity of SC-SGG was unequivocally confirmed by combining the results of nuclear magnetic resonance (NMR) and mass (MS) spectrometry. MS analysis showed a single peak (655.7) corresponding to the molecular mass of the target SC-SGG (calculated for C_31_H_59_O_12_S^−^: 655.4) (Figure 2B). In addition, ^1^H-NMR analysis of the final SC-SGG (**1**) confirmed that the sulfation has occurred at the 3′ position of the galactose structure, as shown by the downfield shift of the signals of the H-3 proton of galactose from ~3.5 (Figure 2C) to ~4.2 ppm (Figure 2C). This was due to the electron-attracting effect of the inserted sulfate group.

### 2.2. Differential Solubility in an Aqueous Environment Between SC-SGG and SGG

**Figure 3:**
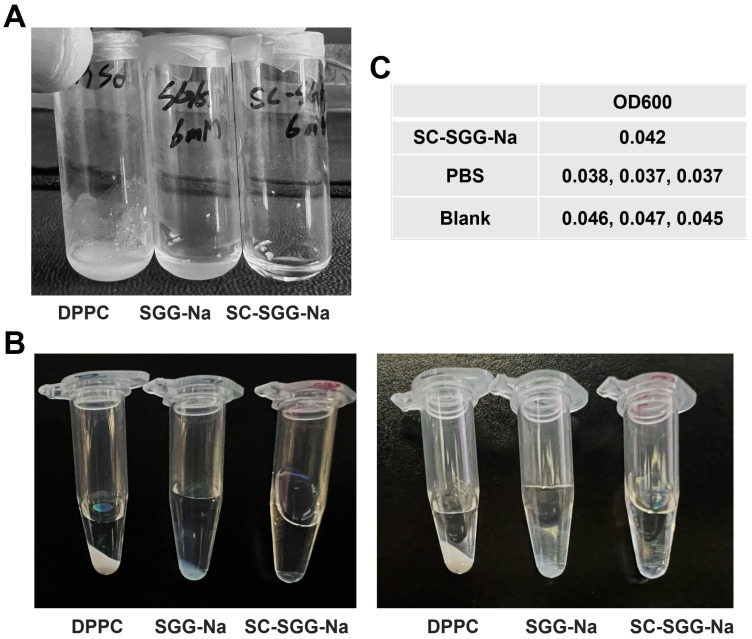
SC-SGG is soluble in an aqueous buffer, whereas SGG and DPPC are not. SC-SGG-Na and SGG-Na, as well as DPPC, each in the powder form were weighed and added into PBS buffer containing 1% DMSO, placed in a small glass vial, so that the final concentration of each lipid was 6 mM. (**A**) SC-SGG-Na was obviously soluble in 1% DMSO in PBS, whereas SGG-Na and DPPC at the same concentration were not. The turbidity of SGG-Na and DPPC suspensions in the buffer was apparent. (**B**) When SC-SGG-Na solution, SGG-Na suspension and DPPC suspension, each in a clear Eppendorf tube, were centrifuged at 14,000× *g* for 15 min, there was no pellet in the SC-SGG-Na solution, whereas a pellet was observed for both the DPPC suspension and SGG-Na suspension. Two picture sets of the same three tubes taken at different angles are shown. (**C**) The solubility of SC-SGG-Na in 1% DMSO in PBS was further confirmed by measurement of OD600 in a microtiter plate. The SC-Na had an OD600 of the same range as PBS placed in adjacent wells and neighboring blank wells.

A comparative study on the solubility of SC-SGG versus SGG in an aqueous buffer revealed that SC-SGG-Na (6 mM) was soluble in 1% DMSO in PBS, whereas SGG-Na at the same concentration was not. Dipalmitoylphosphatidylcholine (DPPC, having two C16 acyl chains but no net negative charge) of the same concentration was also included in the experiments. As shown in Figure 3A, the solution of SC-SGG-Na in 1% DMSO in PBS was clear. In contrast, SGG-Na and DPPC remained undissolved in the same buffer and the suspension was obviously turbid. Further centrifugation at a high speed of the SC-SGG-Na solution, SGG-Na suspension and DPPC suspension, all in 1% DMSO in PBS, resulted in the formation of the lipid pellet for SGG-Na and DPPC, but not for SC-SGG-Na (Figure 3B). This additionally indicated that only SC-SGG was soluble in the aqueous environment. The lipid pellet appeared to be larger for the DPPC suspension. This could arise from the fact that DPPC does not have any net charge, whereas SGG-Na is ionized into SGG with a negative charge on the sulfate group. The complete solubility of SC-SGG in 1% DMSO in PBS was finally confirmed by its baseline value of OD600 as measured in a microtiter plate, i.e., of the same range as that of PBS and blank wells (Figure 3C). Indeed, the substitution of the C16 natural acyl chain with a C6-hexanoyl chain made SC-SGG fully soluble in an aqueous environment, thus making compound **1** (SC-SGG) a good candidate for sperm–egg interaction studies.

### 2.3. Inhibitory Effects of SC-SGG on Mouse In Vitro Fertilization

**Figure 4:**
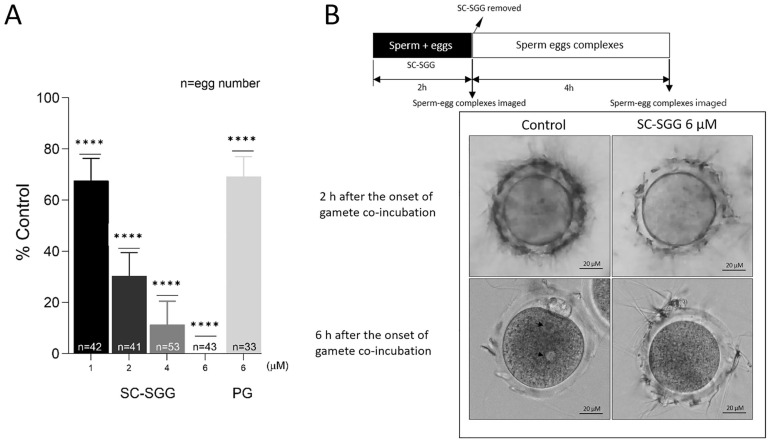
(**A**) Inhibition of mouse in vitro fertilization by SC-SGG present during the gamete co-incubates. Mouse mature eggs (10–20) were co-incubated with capacitated mouse sperm (1 million) in a culture plate in 500 µL of KSOM-0.1% BSA containing various concentrations of SC-SGG (0, 1, 2, 4 and 6 µM) or PG (6 µM). Gamete co-incubates treated with 0 µM SC-SGG (untreated) served as controls. The gametes were co-incubated (37 °C, 5% CO_2_) for 2 h to allow sperm to bind to the eggs at the zona pellucida. At the end of this time period, sperm–egg complexes were removed from the culture plate well and washed successively in 50 µL droplets of KSOM-0.1% BSA + amino acid (both essential and non-essential) supplements (KSOM-0.1%BSA-AA) to remove loosely bound sperm and their images were microscopically recorded. The sperm–egg complexes were further cultured in the 50 µL droplet for another 4 h, and each egg was then assessed for the presence of two pronuclei, as the indication of successful fertilization. The in vitro fertilization rates from three replicate experiments were reported as means ± SDs of percent control (untreated gamete co-incubates). ANOVA was performed on the raw data (% eggs fertilized) of all samples. **** denotes *p* < 0.0001. n = total number of eggs in all replicate experiments. (**B**) Images of sperm–egg complexes from gamete co-incubates (with or without treatment with 6 µM SC-SGG) at 2 h after exposure to SC-SGG, and at 6 h (4 h after sperm-complexes were cultured in fresh KSOM-0.1% BSA-AA). Note that the arrow heads mark the two pronuclei in an egg from the control sample.

When SC-SGG was present in the co-incubates of mature eggs and capacitated sperm, the in vitro fertilization rates were decreased in a concentration-dependent manner. At 1, 2 and 4 µM of SC-SGG, the IVF rate was 68%, 30% and 10% of the control values (untreated with SC-SGG), respectively. The IVF rate then became zero in the presence of 6 µM of SGG. In contrast, 6 µM of PG (with the structure shown in Figure 1) inhibited the IVF rate to only 68% control, a comparable level to that obtained with 1 µM SC-SGG treatment (Figure 4A). However, DMSO (0.01%), added into the gamete co-incubates, had no inhibitory effects on IVF.

Significantly, the ZP of eggs, which were treated with 6 µM SC-SGG for two hours during co-incubation with capacitated sperm, showed a reduced number of bound sperm as compared with the ZP of eggs in the control co-incubates (untreated) (Figure 4B, top row images). In the untreated control sample, during the next four hours of culturing sperm–egg complexes in fresh medium, the ZP-bound sperm would have further penetrated the ZP and one sperm would have bound to and fused with the egg plasma membrane, followed by the incorporation of that specific sperm into the egg proper. The cortical reaction immediately took place with a release of hydrolytic enzymes to modify the ZP in such a manner that it was not receptive to sperm binding [4]. As a result, the number of sperm bound to the ZP was minimal at the end of the 4 h sperm–egg complex incubation in fresh medium (6 h after the initial gamete co-incubation) (Figure 4B, bottom row, left image). The sperm entered then became a male pronucleus concurrently with the formation of a female pronucleus, and thus the fertilized egg appeared with two pronuclei (Figure 4B, bottom row, left image). In contrast, at the end of 6 h after the initial gamete co-incubation, the number of sperm bound to the ZP of the eggs that were treated with 6 µM SC-SGG was still similar to that observed two hours post-gamete co-incubation (Figure 4B, bottom row, right images).

**Figure 5:**
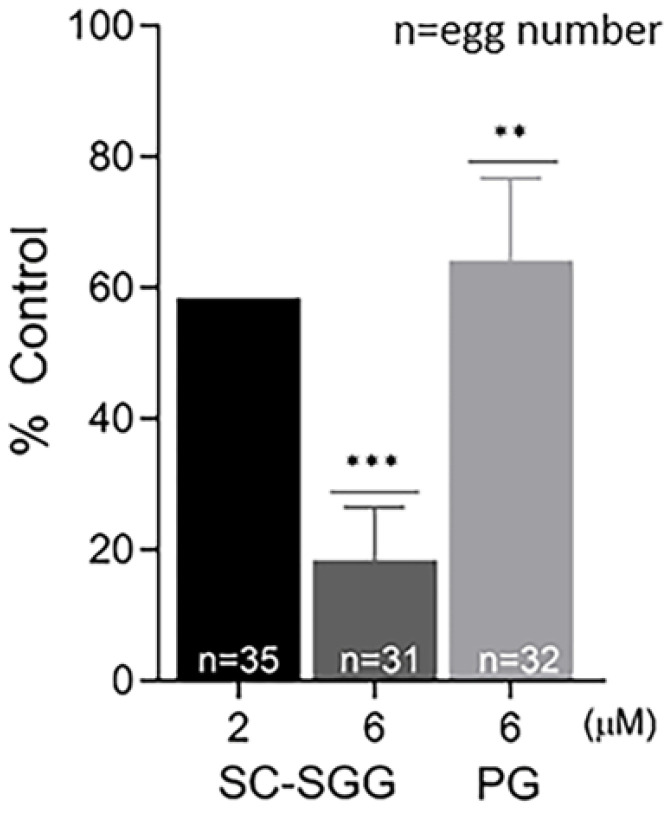
Pretreatment of sperm with SC-SGG reduces their ability to fertilize eggs. Capacitated Percoll gradient-centrifuged (PGC) sperm resuspended in KRB-0.03% BSA (>90% motile with hyperactivated motility patterns) at 10 million sperm/mL were treated with 0, 2 and 6 µM SC-SGG or 6 µM PG (37 °C, 5% CO_2_, 15 min). Capacitated PGC sperm treated with 0 µM SC-SGG (untreated) served as controls. After removing excess SC-SGG and PG from sperm by low-speed centrifugation, one million of these sperm were added into 500 µL of KSOM-0.03% BSA in a culture plate containing untreated eggs (10–15 eggs). The gamete co-incubation duration (2 h) in the culture plate, the washing of excess unbound sperm from the sperm–egg complexes and further culturing (another 4 h) of the sperm–egg complexes in droplets of KSOM-0.1% BSA-AA were performed following the same procedures as described in Figure 4. The presence of two pronuclei in each egg, as the indication of successful fertilization, was then assessed. The in vitro fertilization rates of the gamete co-incubates with sperm pretreated with 6 µM SC-SGG or 6 µM PG were reported as means ± SDs of percent control from three replicate experiments. ANOVA was performed on the raw data (% eggs fertilized) among untreated control samples and those with sperm pretreated with 6 µM SC-SGG or 6 µM PG. **, *** denote *p* < 0.01 and 0.001, respectively. However, experiments with sperm pretreated with 2 µM SC-SGG were performed twice, and the average of their percent control was reported. n = total number of eggs in all replicate experiments.

In order to determine whether the inhibitory effects of SC-SGG and PG on mouse in vitro fertilization were due to impairment to the eggs or sperm, eggs or sperm were pretreated with SC-SGG or PG prior to co-incubation with sperm or eggs, respectively. Pretreatment of sperm with 2 and 6 µM SC-SGG decreased the IVF rate to 59 and 18% control, respectively. Pretreatment of sperm with 6 µM PG also decreased the IVF rate, but not as strong as the effect of SC-SGG of the same concentration, i.e., only to 62% (Figure 5). Interestingly, the inhibitory effects of SC-SGG and PG on sperm fertilizing ability were not due to a decrease in sperm motility. The motility of sperm pretreated with 0 (control), 2 or 6 µM SC-SGG or 6 µM PG was comparable with each other (Supplementary Video S1). Treatment of sperm with 0.01% DMSO did not change sperm motility either.

**Figure 6:**
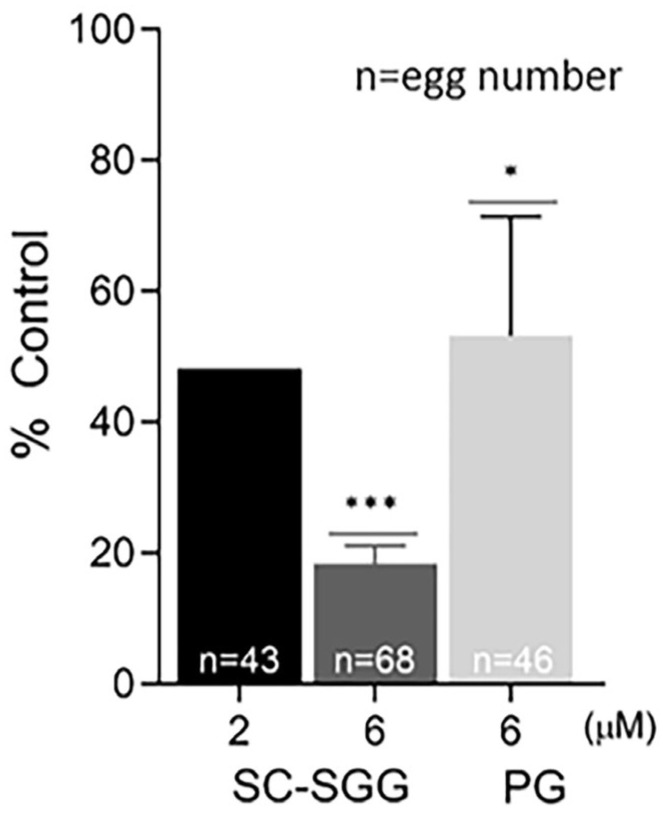
Pretreatment of eggs with SC-SGG reduces their fertilization rates. Mature eggs (15–25) in a droplet of KSOM-0.03% BSA were treated (37 °C, 5% CO_2_, 1 h) with 0, 2 and 6 µM SC-SGG or 6 µM PG. Eggs treated with 0 µM SC-SGG (untreated) served as controls. Eggs were then washed successively in 3 KSOM-0.03% BSA droplets to remove excess unbound SC-SGG or PG. They were subsequently added into 500 µL of KSOM-0.03% BSA in a culture plate containing 1 million capacitated PGC sperm. The gamete co-incubation duration (2 h) in the culture plate, the washing of excess unbound sperm from the sperm–egg complexes and further culturing (another 4 h) of the sperm–egg complexes in droplets of KSOM-0.1% BSA-AA were performed following the same procedures as described in Figure 4. The presence of two pronuclei in each egg, as the indication of successful fertilization, was then assessed. The in vitro fertilization rates of the gamete co-incubates with eggs pretreated with 6 µM SC-SGG or 6 µM PG were reported as means ± SDs of percent control from three replicate experiments. ANOVA was performed on the raw data (% eggs fertilized) among untreated control samples and those with eggs pretreated with 6 µM SC-SGG or 6 µM PG. *, *** denote *p* < 0.05 and 0.001, respectively. However, experiments with eggs pretreated with 2 µM SC-SGG were performed twice, and the average of their percent control was reported. n = total number of eggs in all replicate experiments.

**Figure 7:**
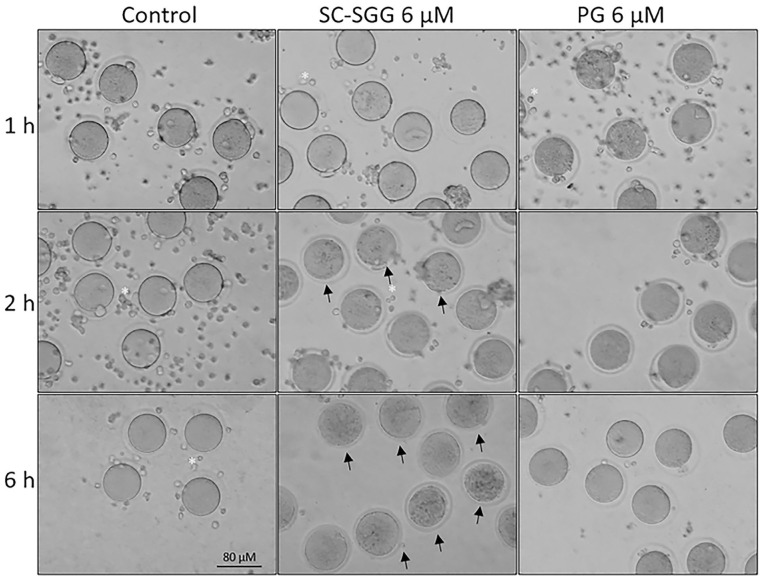
Morphology of control eggs and eggs treated with SC-SGG or PG for various time periods. Mature eggs (15–20) in a droplet of KSOM-0.03% BSA were treated (37 °C, 5% CO_2_) with 0 and 6 µM SC-SGG or 6 µM PG for 1, 2 and 6 h. Eggs treated with 0 µM SC-SGG served as controls. At the end of each treatment time, eggs were washed successively in 3 KSOM-0.03% BSA droplets to remove excess unbound SC-SGG or PG, and their morphology was microscopically recorded. Small cells (*) surrounding the eggs were cumulus cells, which were dissociated from the eggs by hyaluronidase treatment of cumulus–oocyte complexes. Arrows in the bottom-middle panel signify the degenerated feature of eggs treated with SC-SGG for 6 h. These eggs contained dark, clumped elements in the cytoplasm. Data presented were representative of three replicate experiments.

Pretreatment of eggs with SC-SGG or PG showed inhibitory effects on the IVF rate with a similar trend to those observed in the sperm pretreatment experiments. Eggs pretreated with 2 and 6 µM SC-SGG had the IVF rate of 48 and 18% control, respectively, whereas the IVF rate of eggs pretreated with 6 µM PG was 52% (Figure 6). Notably, the inhibitory effects of SC-SGG on the egg fertilization rate could be attributed to its blocking of the sperm surface ligand on the egg plasma membrane. SLIP1 (sulfolipid-immobilizing protein 1, which was identified to be arylsulfatase A), is present on the egg plasma membrane [24], and the interaction between egg plasma membrane SLIP1 and surface SGG on the acrosome-reacted sperm is likely the basis of how sperm and egg plasma membranes bind to each other, the step preceding the sperm–egg plasma membrane fusion and the incorporation of one sperm into the egg proper, a hallmark of fertilization. The egg plasma membrane SLIP1 would be saturated with SC-SGG upon incubation of eggs with SC-SGG, and the egg SLIP1 would no longer be able to interact with the surface SGG of the acrosome-reacted sperm, thus resulting in a decrease in the IVF rate of SC-SGG treated eggs. In addition, the supersaturation of egg SLIP1 with SC-SGG may result in downstream impairment of the egg structure. Dark and clumped subcellular elements, features of egg degeneration, were observed in eggs treated with 6 µM SC-SGG at 2 and 6 h after the initial gamete co-incubation. In contrast, eggs treated with 6 µM PG appeared normal, like the control eggs (Figure 7).

## 3. Discussion

The synthetic strategy for SC-SGG described herein was very robust and efficient, smoothly providing the target compound in three steps starting from compound **2**. This feasible protocol is one of the few reported in the literature for the synthesis of SGG derivatives [26,27,28]. It can also be used by employing a select acylating agent for the preparation of the SGG molecular species, which is prevalently found in body tissues, i.e., with C16 alkyl and C16 acyl chains.

The synthesized SC-SGG was fully soluble in an aqueous environment (1% DMSO in PBS in this report), while SGG with the C16 acyl chain, chemically synthesized by the same procedure (Supplementary Material Part 2) was not soluble (Figure 3). The availability of SC-SGG allowed us to determine whether this SC-SGG had inhibitory effects on mouse IVF in the aqueous environment. We demonstrated that SC-SGG, when present for two hours in the mouse gamete co-incubates, had inhibitory effects on IVF in a concentration-dependent manner, and at 6 µM SC-SGG, the IVF rate was zero. Imaging of the sperm–egg complexes after two hours of gamete co-incubation in the presence of 6 µM SC-SGG indicated that the sperm binding to the egg ZP was much reduced, as compared with the corresponding binding of the control sperm–egg complexes. Four hours of additional co-incubation in a fresh medium without SC-SGG showed that the number of sperm bound to the ZP of the sperm–egg complexes (treated with 6 µM SC-SGG in the preceding 2 h) remained similar to that at the end of 2 h gamete co-incubation. This was in contrast to that observed in control sperm–egg complexes, where the majority of sperm bound to the ZP, as seen at the end of the 2 h gamete co-incubation, fell off from the ZP due to the modification of the ZP structure by hydrolytic enzymes released from the cortical reaction, taking place immediately after the sperm–egg plasma membrane fusion and the entry of one sperm into the egg proper [1,3,4]. The results indicated that SC-SGG interfered with the initial sperm–ZP interaction and the adverse effect caused by this interference was not reversible, thus affecting the downstream events, the sperm–egg plasma membrane interaction/fusion and sperm incorporation into the egg. Overall, the IVF rates of SC-SGG-treated gametes decreased. These inhibitory effects of SC-SGG could have been on sperm, eggs or both.

The sperm or egg pretreatment experiments indeed indicated that SC-SGG had adverse effects on sperm and eggs, each of which led to decreases in the IVF rates. However, the adverse effects of SC-SGG on sperm alone or eggs alone were not as severe as the combined effects on the sperm–egg co-incubates. At 2 µM SC-SGG, the IVF rates of pretreated sperm were about 60% of the control and those of pretreated eggs were also about 60% of the control, whereas the combined effects of this SC-SGG concentration on sperm–egg co-incubates led to the IVF rates of close to 30% of the control. In a similar vein, the combined effects of 6 µM SC-SGG on sperm–egg co-incubates led to zero IVF rates, whereas its effects on sperm alone or egg alone led to the IVF rates of close to 20% control in both cases.

Interestingly, sperm pretreated with 6 µM SC-SGG still remained motile, like the control sperm. The adverse effects of SC-SGG on sperm may come from the insertion of SC-SGG into the sperm head site where SGG is localized, i.e., at the convex ridge of acrosome-intact sperm or the inner acrosomal membrane and the plasma membrane of the post-acrosomal region of acrosome-reacted sperm [10,17]. Although the acyl chain of SC-SGG only contains six carbons, its alkyl chain is still of a C16 chain with the ability to hydrophobically interact with either the C16 acyl or C16 alkyl chain of SGG on sperm. The insertion of an externally added SGG analog (galactose(3-sulfate)-β1-1′[*N*-lissamine rhodaminyl)-12-aminododecanoyl-sphingosine: SGalCer (C12-LRh)) into the sperm head where SGG is localized has been documented [30]. Acrosome-intact sperm with SC-SGG inserted in the sperm head convex ridge (the site of ZP binding) would have a modified plasma membrane, which may not bind the ZP well. These SC-SGG-inserted sperm may or may not undergo acrosome reaction and if they do not, the sperm–egg plasma membrane binding and fusion is unable to take place, and thus fertilization cannot be completed. Although the SC-SGG inserted sperm could undergo acrosomal exocytosis and the inserted SC-SGG was removed from acrosome reacted sperm, the remaining SC-SGG in the surrounding could still incorporate into the inner acrosomal membrane and the post-acrosomal plasma membrane, and in the same vein, these SG-SGG-inserted acrosome-reacted sperm may not be able to bind and fuse with the egg plasma membrane for the completion of fertilization.

In addition to the direct binding of SGG to the ZP, an SGG-binding protein, ARSA, which is colocalized with SGG on the surface of both acrosome intact and acrosome reacted sperm, possesses specific affinity for the ZP [15,16,17,18]. Therefore, sperm SGG and ARSA likely act synergistically in sperm–ZP binding [9,12]. The interaction between SGG and ARSA on the sperm surface is with a high affinity and occurs through the binding of SGG to a cleft on the ARSA surface [15,17]. While the externally added SC-SGG may not be able to disrupt the already existing SGG–ARSA conjugation on the sperm surface, having a short acyl chain, SC-SGG may enter and bind to the enzyme active site pocket of ARSA [25]. This SGG binding may lead to a change in the ARSA contour and its interaction with the adjacent SGG on the sperm surface may be decreased, and thus the synergistic activity of SGG and ARSA in the sperm–ZP binding could be reduced. This could be an additional cause on how SC-SGG diminished the sperm fertilizing ability. Although the molecular interaction between SGG and ACRBP is not known, SC-SGG may decrease the ZP binding ability of ACRBP in a similar manner to that proposed for ARSA.

The decreases in IVF rates of eggs pretreated with SC-SGG could be from the saturation of the binding site of sperm SGG on egg plasma membrane SLIP1 by the added SC-SGG. Furthermore, SC-SGG may induce its impairment on the egg structure because of the downstream transduction following its binding to the egg SLIP1, or due to an alternative signaling pathway unrelated to SLIP1 interaction. The egg impairment was apparent by the presence of dark and clumped subcellular elements in the egg proper, which were characteristic of eggs that are being degenerated.

In addition to the direct impairment to sperm and eggs, SC-SGG when present in the sperm–egg co-incubates could act as a competitive inhibitor of the interaction between SGG on the acrosome-intact sperm and the ZP [10] and between SGG on the acrosome-reacted sperm and SLIP1 on the egg plasma membrane [17,24]. Both lines of inhibition would result in a decrease in IVF rates when SC-SGG was present in the gamete co-incubates.

As expected, PG did not have a marked effect, as seen with SC-SGG, on the IVF rates when present in the gamete co-incubates or when it was used to pretreat sperm or eggs. PG has a glycerol backbone with an ether C16 hydrocarbon chain. While both of these structural moieties exist in SC-SGG, PG does not contain galactose sulfate and the ester linkage, which are part of SC-SGG (see their structures in Figure 1). In particular, galactose sulfate is likely important for SC-SGG to exert its high inhibitory effects on sperm–egg interactions. The importance of this galactose sulfate moiety for the activity of SGG to bind to the egg ZP has been previously documented [9,10]. Nonetheless, PG still inhibited IVF rates to a certain extent and this may be due to its random incorporation into the plasma membrane of sperm and eggs, thus interfering in the sperm–egg plasma membrane interaction/fusion.

Overall, our results in the SC-SGG study presented herein confirms our previous findings that SGG plays a role in sperm–egg interaction/fertilization. Our previous study, however, employed SGG unilamelllar liposomes to demonstrate their direction interaction with the ZP in vitro [10]. The use of SGG unilamellar liposomes as an inhibitor of fertilization in vivo is, however, rather limited due to the insolubility of the liposomes and thus difficulty in their specific delivery to the target gametes in the oviduct. SC-SGG is more aqueous soluble as confirmed by its inhibitory effects on IVF as shown herein. It can be packed into nanoparticles with the surface functionalization to contain a protein(s) with affinity for the egg ZP, such as oviductin, for target delivery to the ovulated eggs in the oviduct [33]. Such nanoparticle drug delivery has been described for target tissues in the female reproductive tract [34]. Potentially, SC-SGG can be developed into a non-hormonal contraceptive, which is expected to have less side effects than hormonal contraceptives operated by systemic interference of hormonal levels in the body [35,36].

## 4. Materials and Methods

### 4.1. Chemical Synthesis of SC-SGG

All reagents and chemicals used in the chemical synthesis of the intermediates and final compounds were purchased from Merck KGaA (Darmstadt, Germany) and used without any further purification. All the reactions were performed under Argon atmosphere with the use of anhydrous solvents that were dried before use. Dichloromethane (DCM) was freshly distilled from CaH_2_ prior to use. Methanol (MeOH) was dried over activated molecular sieves. Reaction monitoring was performed by thin-layer chromatography (TLC) using Merck silica gel on silica gel 60 F-254 TLC-PET foils with detection by spraying with 50% H_2_SO_4_ solution or with phosphomolybdate-based reagent and heating to 110 °C. The products of the reactions were purified by flash chromatography [37]. Flash column chromatography was performed using a high-purity grade silica gel (SiO_2_, high-purity grade (9385), pore size 60 Å, 230–400 mesh particle size) by Merck. The purity of all synthetized compounds was determined by nuclear magnetic resonance (NMR) analysis and mass spectrometry (MS). NMR analysis was performed using a 500 MHz Bruker FT-NMR AVANCE DRX500 spectrometer (pulsed field gradient, reverse broadband probe, ^1^H at 500.13 MHz and ^13^C at 125.77 MHz) at a sample temperature of 298 K.

The chemical shift in each signal is given in parts per million (ppm) relative to residual CHCl_3_ fixed at 7.28 ppm for CDCl_3_, and 7.27 ppm for CDCl_3_/CD_3_OD solutions for ^1^H NMR spectra and relative to CDCl_3_ fixed at 77.0 ppm (central line) for CDCl_3_ ^13^C-NMR spectra. Coupling constants (*J*) are given in Hertz (Hz). The multiplicity observed in the ^1^H-NMR spectra for each signal is labeled as s (singlet), d (doublet), t (triplet), dd (doublet doublet), and m (multiplet). Mass spectra were recorded in negative- or positive-ion electrospray (ESI) mode on a Thermo Quest Finnigan LCQTMDECA ion trap mass spectrometer, equipped with a Finnigan ESI interface; sample solutions were injected with a ionization spray voltage of 4.5 kV or 5.0 kV (positive- and negative-ion mode, respectively), a capillary voltage of 32 V or −15 V (positive- and negative-ion mode, respectively), and capillary temperature of 250 °C; em = exact mass. Optical rotations were measured at room temperature with a Perkin-Elmer 241 polarimeter (589 nm, D line from N lamp). The logarithm of n-octanol–water partition coefficient (logP) has been used as an indicator of the lipophilicity of SC-SGG, and it was calculated with the QikPro module from the MAESTRO suite of Schrodinger (version 2023-4). LogD was calculated with the LogD calculation suite of the ChemAxon modeling package. Compound **2** (3-*O*-hexadecyl-1-*O*-(2,3,4,6-tetra-*O*-benzyl-β-d-galactopyranosyl)-*sn*-glycerol) was used as the starting compound in the synthesis of SC-SGG. It was synthesized according to the procedure previously published by Lindberg, and its analytical data are consistent with those reported in the reference [31]. ^1^H-NMR, ^13^C-NMR and MS spectra of the newly synthesized compounds are reported in Supplementary Material Part 1.

#### Procedures for the Synthesis of Short-Chain SGG (SC-SGG, Compound **1**)

The scheme of this synthesis is pictorially shown in Figure 2A.

- *Synthesis of 1-O-Hexadecyl-2-O-hexanoyl-3-O-(2,3,4,6-tetra-O-benzyl-β-d-galactopyranosyl)-sn-glycerol (compound* **3***)*

To a solution of compound **2** (0.09 g, 0.11 mmol) in dry DCM (10 mL) under Ar atmosphere were added 1-ethyl-3-(3-dimethylaminopropyl)carbodiimide (EDCI, 0.084 g, 0.44 mmol), 4-dimethylaminopyridine (DMAP, 0.028 g, 0.22 mmol) and caproic acid (0.040 mL, 0.33 mmol). The reaction mixture was refluxed for 2.5 h. Then, it was diluted with DCM and washed with NaHCO_3_-saturated solution (2 × 10 mL). The organic layer was dried over Na_2_SO_4_, filtered and concentrated under reduced pressure. Compound **3** was obtained pure after flash column chromatography (hexane/ethyl acetate 9:1) as a colorless oil (0.074 g, 74%). R_f_: 0.6 (petroleum ether/EtOAc, 8:2); ${\left[\alpha \right]}_{D}^{20}$ = −1.22 (*c* 0.5 in CHCl_3_); ^1^H-NMR (CDCl_3_) δ 7.43–7.24 (m, 20H, arom.), 5.24–5.18 (m, 1H, H-b), 4.99–4.91 (m, 2H, OCH_2_Ph), 4.81–4.70 (m, 3H, OCH_2_Ph), 4.64 (d, 1H, *J* = 11.7 Hz, OCH_2_Ph), 4.50–4.40 (m, 2H, OCH_2_Ph), 4.36 (d, 1H, *J*_1,2_ = 7.7 Hz, H-1), 4.04 (dd, 1H, *J*_a,a’_ = 10.8 Hz, *J*_a,b_ = 4.9 Hz, H-a), 3.91 (d, 1H, *J*_3,4_ = 2.6 Hz, H-4), 3.82 (dd, 1H, *J*_1,2_ = 7.7 Hz, *J*_2,3_ = 7.9 Hz, H-2), 3.72 (dd, 1H, *J*_a,a’_ = 10.8 Hz, *J*_a’,b_ = 4.8 Hz, H-a’), 3.67–3.57 (m, 4H, 2 H-6 and 2 H-c), 3.56–3.50 (m, 2H, H-3 and H-5), 3.48–3.33 (m, 2H, 2 H-d), 2.35–2.23 (m, 2H, CH_2_CO), 1.69–1.47 (m, 4H, C*H*_2_CH_2_CO and O CH_2_C*H*_2_), 1.40–1.18 (m, 30H), 0.94–0.85 (m, 6H, 2 CH_3_); ^13^C-NMR (CDCl_3_) δ 173.2 (C=O), 138.7, 138.6, 138.5, 137.9, 128.4–127.4 (20 C), 104.3 (C-1), 82.1 (C-3), 79.3 (C-2), 75.0 (CH_2_Ph), 74.5 (CH_2_Ph), 73.5 (3C, CH_2_Ph, C-4 and C-5), 73.1 (CH_2_Ph), 71.5 (C-d), 71.3 (C-c), 69.3 (C-6), 68.7 (C-c), 68.2 (C-a), 34.3 (CH_2_CO), 31.9, 31.2, 29.7–29.3 (11 C), 26.0, 24.6 (*C*H_2_CH_2_CO), 22.6, 22.2, 14.1, 13.9; ESI-MS (positive-ion mode): *m*/*z* for C_59_H_84_O_9_ (em: 936.6): 959.9 [M + Na^+^].

- *Synthesis of 1-O-Hexadecyl-2-O-hexanoyl-3-O-(β-d-galactopyranosyl)-sn-glycerol (compound* **4***)*

Palladium catalyst over carbon (Pd/C, 0.050 g) was added to a solution of compound **3** (0.065 g, 0.069 mmol) in methanol (4 mL) and ethyl acetate (2 mL). The reaction flask was flushed three times with hydrogen, and the reaction was stirred, under hydrogen atmosphere, for 3 days. The mixture was filtered through a celite pad, then the filtrated solution was evaporated under reduced pressure. Compound **4** was obtained pure after flash column chromatography (DCM/MeOH, 95:5) as an oil (0.031 g, 78%). R_f_: 0.4 (DCM/MeOH, 9:1); ${\left[\alpha \right]}_{D}^{20}$ = −13.7 (*c* 1 in CHCl_3_); ^1^H-NMR (CD_3_OD:CDCl_3_, 1:1) δ 5.24–5.15 (m, 1H, H-b), 4.22 (d, 1 H, *J*_1,2_ = 7.4 Hz, H-1), 3.95 (dd, 1H, *J*_a,a’_ = 10.9 Hz, *J*_a,b_ = 5.9 Hz, H-a), 3.86 (*br* d, 1H, *J*_3,4_ = 3.3 Hz, H-4), 3.79 (dd, 1H, *J*_5,6a_ = 6.6 Hz, *J*_6a,6b_ = 11.4 Hz, H-6a), 3.77–3.70 (m, 2H, H-6b and H-a), 3.66–3.57 (m, 2H, 2 H-c), 3.57–3.39 (m, 5H, H-2, H-3, H-5 and 2 H-d), 2.34 (t, 2H, *J* = 7.5 Hz, 2 CH_2_CO), 1.68–1.58 (m, 2H, C*H*_2_CH_2_CO), 1.58–1.51 (m, 2H, OCH_2_C*H*_2_), 1.41–1.16 (m, 28H), 0.97–0.81 (m, 6H, 2 CH_3_); ^13^C-NMR (CD_3_OD/CDCl_3_, 1:1) δ 174.0 (C=O), 103.9 (C-1), 75.1 (C-5), 73.4 (C-3), 71.6 and 71.5 (C-b and C-d), 71.1 (C-2), 69.2 (C-c), 68.7 (C-4), 68.1 (C-a), 61.1 (C-6), 34.1 (CH_2_CO), 31.7, 31.0, 29.5–29.1 (11 C), 25.8, 24.4 (*C*H_2_CH_2_CO), 22.4, 22.1, 13.6, 13.4; ESI-MS (positive-ion mode): *m*/*z* for C_31_H_60_O_9_ (em: 576.42): 599.23 [M + Na]^+^, 1175.27 [2M+Na]^+^.

- *Synthesis of 1-O-Hexadecyl-2-O-hexanoyl-3-O-[3-O-(sodium oxysulfonyl)-β-d-galactopyranosyl)-sn-glycerol (compound* **1***, SC-SGG)*

Compound **4** (0.024 g, 0.042 mmol) was dissolved in dry methanol (4 mL) under Ar atmosphere. Dibutyltin oxide (Bu_2_SnO, 0.016 g, 0.063 mmol) was added, and the reaction mixture was left under reflux for 2.5 h. Then, the solvent was evaporated and the crude was dissolved in dry tetrahydrofurane (4 mL). Sulfur trioxide trimethylamine complex (Me_3_N·SO_3_, 0.012 g, 0.084 mmol) was added and the mixture was left under stirring for 2.5 h. The solvent was removed under reduced pressure than the residue was dissolved in CHCl_3_/MeOH, 1:1 and loaded onto a cation exchange resin column (Dowex 50 × 8, Na^+^ form). The mixture was eluted with CHCl_3_/MeOH, 1:1, concentrated and subjected to flash chromatography (CH_2_Cl_2_/MeOH, 9:1) to afford pure compound **1** as an amorphous solid (0.022 g, 76%). R_f_: 0.2 (CH_2_Cl_2_/MeOH, 85:15); ${\left[\alpha \right]}_{D}^{20}$ = +3.8 (*c* 1 in CHCl_3_/MeOH, 1:1); ^1^H-NMR (CDCl_3_/CD_3_OD, 1:1): δ 5.24–5.16 (m, 1H, H-b), 4.33 (d, 1H, *J*_1,2_ = 7.7 Hz, H-1), 4.27 (*br* d, 1H, *J*_3,4_ = 3.2 Hz, H-4), 4.24 (dd, 1H, *J*_2,3_ = 9.7 Hz, *J*_3,4_ = 3.2 Hz, H-3), 3.95 (dd, 1H, *J*_a,a’_ = 10.9 Hz, *J*_a,b_ = 5.8 Hz, H-a), 3.84–3.69 (m, 4H, H-2, 2 H-6 and H-a), 3.67–3.58 (m, 2H, 2 H-c), 3.55 (t, 1H, *J*_5,6a_ = *J*_5,6b_ = 5.8 Hz, H-5), 3.51–3.39 (m, 2H, OC*H*_2_CH_2_), 2.34 (t, 2H, *J* = 7.5 Hz, CH_2_CO), 1.67–1.58 (m, 2H, C*H*_2_CH_2_CO), 1.58–1.49 (m, 2 H, OCH_2_C*H*_2_), 1.38–1.20 (m, 30H,15 CH_2_), 0.93–0.84 (m, 6H, 2 CH_3_); ^13^C-NMR (CDCl_3_/CD_3_OD, 1:1): δ 174.0 (C=O), 103.6 (C-1), 80.5 (C-3), 74.7 (C-5), 71.6 (C-b), 71.5 (C-d), 69.3 and 69.2 (C-2 and C-c), 68.0 (C-a), 67.1 (C-4), 61.2 (C-6), 34.1 (CH_2_CO), 31.7, 31.0, 29.4–29.1 (11 C), 25.8, 24.4, 22.4, 22.1, 13.5, 13.3; ESI-MS (negative-ion mode): *m*/*z* for C_30_H_59_NaO_12_S (em: 678.36): 655.67 [M–Na]^−^, 1333.4 [2M–2Na + 23]^−^.

### 4.2. Differential Solubility of SC-SGG Versus SGG in 1% Dimethylsulfoxide (DMSO) in PBS

#### 4.2.1. Chemicals

SC-SGG-Na (MW: 678) was chemically synthesized as described in Figure 2. SGG-Na (MW: 818) was also chemically synthesized following the same procedure as for SC-SGG-Na, except that palmitic acid instead of caproic acid was used in the acylation step (see Supplementary Material Part 2). DPPC (1,2-dipalmitoyl-sn-glycero-3-phosphocholine) (MW: 734) was purchased from Sigma-Aldrich (St. Louis, MO, USA). DPPC was included in this study as it has two C16 hydrocarbon chains like SGG, but it contains no net negative charge (in contrast to SGG).

#### 4.2.2. Assessment of the Solubility of SC-SGG-Na, SGG-Na and DPPC in 1% DMSO in PBS

Each compound in the powder form was weighed and added into the PBS buffer containing 1% DMSO, and placed in small glass vials so that the final concentration of each lipid was 6 mM. The volume of DPPC in the suspension was 300 µL, of SGG-Na in suspension was 350 µL and of SC-SGG-Na in solution was 350 µL. The clarity/turbidity of each lipid tube was assessed by eyeing and the tubes were photographed. The DPPC suspension, SGG-Na suspension and SC-SGG solution were each then transferred into a clear Eppendorf tube, and subjected to centrifugation at 14,000× *g* for 15 min to determine whether there was pellet formation at the bottom of the tube. Finally, the SC-SGG-Na solution (50 µL) was placed in a well of a microtiter plate along with PBS (50 µL) in three adjacent wells. OD at 600 nm (revealing turbidity or precipitated materials) was read on SC-SGG-Na, PBS as well as neighboring blank wells by a SpectraMax Plus 384 plate reader (Molecular Devices, San Jose, CA, USA).

### 4.3. Effects of SC-SGG and Palmitylglycerol on Mouse Sperm Motility, Sperm–Zona Pellucida (ZP) Interaction and In Vitro Fertilization (IVF)

#### 4.3.1. Chemicals

SC-SGG sodium salt (MW: 678) was synthesized as described in this report. Palmitylglycerol (PG, 1-*O*-hexadecyl-*sn*-glycerol; MW: 316.5) was purchased from Avanti Polar Lipids (Cat. No. 999971P) (Birmingham, AL, USA). Other chemicals used were from Sigma-Aldrich (Oakville, ON, Canada) unless otherwise noted. The catalog number of bovine serum albumin used for supplementing KRB and KSOM medium was Sigma A3311.

#### 4.3.2. Animals

CD-1 male mice (~5 months old) and CF-1 female mice (~2 months old) were obtained from Charles River Laboratories (Senneville, QC, Canada). All mice were kept in a temperature-controlled (22.5 °C) room with a 12 h light (7 a.m. to 7 p.m.) and 12 h dark (7 p.m. to 7 a.m.) photoperiod. They were fed ad libitum with Purina rodent chow and water. Handling and use of these mice for experiments were conducted according to the Canadian Council on Animal Care guidelines, with approval of our protocol (#2568) by the University of Ottawa Animal Care committee, which also endorsed the use of ARRIVE checklists and guidelines. Mice were sacrificed by cervical dislocation. The epididymis and vas deferens were dissected from the males for the sperm collection, whereas the oviducts were removed from the females for the retrieval of mature eggs.

#### 4.3.3. Preparation of SC-SGG and PG Solutions for Treatment of Gametes

Both SC-SGG and PG were dissolved in 1% dimethylsulfoxide (DMSO) to the stock concentration of 6 mM. This stock solution was further diluted serially in medium to lower concentrations, each being 10× of the final working concentration.

#### 4.3.4. Medium

KRB-HEPES was composed of 99.6 mM NaCl, 4.8 KCl, 1.2 mM KH_2_PO_4_, 1.2 mM MgSO_4_, 1.7 mM CaCl_2,_ 5.6 mM glucose, 1 mM sodium pyruvate, 25 mM sodium lactate, 100 IU/mL penicillin G, 0.01% streptomycin sulfate, 0.001% Phenol Red, 4 mM NaHCO_3_, 21 mM HEPES, pH 7.4. KRB-HEPES supplemented with 0.1% polyvinylalcohol (PVA) was used in air for the collection of sperm from the caudal epididymis and vas deferens and handling them through Percoll gradient centrifugation [38].

KRB, composed of the same ingredients as KRB-HEPES, but without HEPES and with 25 mM NaHCO_3_ instead of 21 mM in KRB-HEPES. When equilibrated with 5% CO_2_, the pH of KRB was 7.4. Mouse sperm incubated (37 °C, 1 h, 5% CO_2_) in KRB supplemented with 0.3% bovine serum albumin (BSA) (KRB-0.3% BSA) became fully capacitated, showing hyperactivated motility patterns [39,40], due to the presence of 1.7 mM CaCl_2_, 0.3% BSA and 25 mM NaHCO_3_ in the medium [16,41]. KRB-0.03% BSA was used for incubation of pre-capacitated sperm during their treatment with SC-SGG and PG. The lower concentration of BSA would reduce the propensity of its interaction with the treating lipids (SC-SGG and PG).

KFHM, composed of 95 mM NaCl, 2.5 mM KCl, 0.35 mM KH_2_PO_4_, 0.2 mM MgSO_4,_ 1.7 mM CaCl_2_, 0.2 mM glucose, 0.2 mM sodium pyruvate, 10 mM sodium lactate, 100 IU/mL penicillin G, 0.01% streptomycin sulfate, 4 mM NaHCO_3_, 21 mM HEPES and pH 7.4, was used in air for the collection of mature eggs from the mouse oviduct [42,43].

KSOM was composed of the same ingredients as KFHM but without HEPES and with 25 mM NaHCO_3_ instead of 21 mM in KFHM-HEPES [42,43]. Like KRB, the pH of KSOM was 7.4 when equilibrated with 5% CO_2_. KSOM-0.03% BSA was used for incubation of sperm and eggs during their 2 h treatment with SC-SGG and PG.

KSOM-0.1% BSA supplemented with MEM essential amino acids (Sigma-Aldrich M5550) and MEM non-essential amino acids (Sigma-Aldrich M 7145) were used for 4 h incubation of sperm–egg complexes obtained from the initial 2 h incubation in KSOM-0.03% BSA [42,43].

#### 4.3.5. Preparation of Percoll Gradient-Centrifuged (PGC) Sperm

Sperm were collected from the caudal epididymis and vas deferens into the KRB-HEPES-0.1% PVA medium. The cauda epididymis was cut longitudinally in the middle by a fresh razor blade almost to the bottom, and the sperm fluid was squeezed out from the bottom of the organ towards the top by a pair of forceps. On the other hand, sperm fluid was collected from the vas deferens, which had been cleared from the blood vessel and surrounding fat, by gently squeezing the vas deferens with a pair of forceps along the whole duct. The collected sperm fluids from the cauda epididymis and vas deferens were combined and subjected to Percoll gradient centrifugation, as previously described [38]. Percoll gradient-pelleted sperm were then resuspended in KRB-0.3% BSA (37 °C, 5% CO_2_, 1 h) to induce capacitation. Close to 100% of these capacitated sperm were motile with hyperactivated motility patterns. The density of the sperm suspension was adjusted to 10 million/mL in the same medium before being used in the next step.

#### 4.3.6. Effects of SC-SGG and PG on Motility of Capacitated Mouse Sperm

Capacitated PGC sperm were pelleted by centrifugation (350× *g*, 10 min), resuspended in the same volume of KRB-0.03% BSA and then treated (37 °C, 10 min) with SC-SGG or PG. Five microliters of the stock solution of SC-SGG at 20, 40 and 60 µM was added into 45 µL of the sperm suspension in medium, so that the final concentration of SC-SGG in sperm treatment was 2, 4 and 6 µM, respectively. The effect of PG on sperm motility was assessed only at the final concentrations of 12 and 60 µM. Sperm untreated with either SC-SGG or PG served as controls. Since the 6 mM SC-SGG original stock solution was made in 1% DMSO, the sperm suspension treated with 6, 4 and 2 µM contained DMSO at the concentration of 0.001, 0.0007 and 0.00035%, respectively. To determine whether DMSO had any adverse effects on sperm motility, another 45 µL of the sperm suspension was treated with 5 µL of 0.1% DMSO to give the final DMSO concentration of 0.01%. Ten microliters of the sperm suspension was pipetted as a microdrop onto a glass slide and sperm motility was recorded by video microscopy, using a 10× objective with a Celestron HD Digital Microscope Imager (Torrance, CA, USA). The video recording was performed at 60 frames per second for 20 s.

#### 4.3.7. Collection of Mature Oviductal Eggs

Female mice were induced to superovulate by intraperitoneal (IP) injection of equine chorionic gonadotropin (eCG) (5 IU) a few hours before the start of a dark phase and then IP injection of human chorionic gonadotropin (hCG) (5 IU) 48 h post-eCG injection. Females were euthanized the following morning and cumulus masses containing oocyte cumulus complexes were collected in KFHM from the oviduct and treated with 0.1% hyaluronidase to dissociate cumulus cells from the complexes, thus generating cumulus-free mature eggs, which were kept (37 °C, 5% CO_2_) in a 50 µL droplet of KSOM-0.03% BSA, topped with paraffin oil in a Petri dish, prior to sperm co-incubation.

#### 4.3.8. Effects of SC-SGG and PG on Sperm-Zona Pellucida Interaction and In Vitro Fertilization

On the first set of experiments, SC-SGG or PG were used to treat sperm and eggs during their co-incubation. Sperm and egg co-incubates were prepared as follows.

Capacitated sperm (50 µL) in KRB-0.3% BSA, prepared as described above, were diluted 10× in KSOM to have a density of 1 million/mL in 500 µL of KSOM-0.03% BSA. This sperm suspension was placed in a well of a four-well culture dish into which 20–25 eggs were added and the gametes were co-incubated (37 °C, 5% CO_2_) for 2 h.

The inhibitory effects of SC-SGG on sperm–egg interaction and subsequently on in vitro fertilization were assessed by adding 50 µL of the 10× stock solutions of SC-SGG at the beginning of this 2 h period into each gamete co-incubate, so that the final concentrations of SC-SGG were 2, 4 6, 12 and 60 µM. The effects of PG at the final concentrations of 12 and 60 µM, as well as of 0.01% DMSO, were also investigated following the same protocol. At the end of the 2 h period, sperm–egg complexes were removed from the 4-well culture dish, and washed successively in three 50 µL droplets of KSOM-0.03% BSA and finally pipetted into a 50 µL droplet of KSOM-0.1% BSA supplemented with amino acids (KSOM-0.1% BSA-AA). The sperm–egg complexes were then microscopically visualized, photographed and further cultured (37 °C, 5% CO_2_) for another 4 h. Fertilization was assessed at the end of 4 h by scoring fertilized eggs with two pronuclei [16].

In the second set of experiments, sperm were pretreated with SC-SGG or PG prior to co-incubation with eggs. Capacitated sperm in 50 µL of KRB-0.03% BSA (density: 10 million/mL) were treated (37 °C, 5% CO_2_, 30 min) with 2 µM or 6 µM SC-SGG or 6 µM PG. Excess SC-SGG or PG was removed from the sperm suspension by centrifugation (350 g, 10 min). The pelleted sperm were then resuspended in 500 µL of KSOM-0.03% BSA in the well of a four-well dish to which untreated mature eggs were added. Co-incubation of the gametes and assessment of two pronuclei formation were performed in the same manner as in the experiment above. Capacitated sperm untreated with either SC-SGG or PG and subjected to all steps in parallel with the treated sperm served as controls.

In the third set of experiments, eggs were pretreated with SC-SGG. The oviductal eggs collected were moved by pipetting into 50 µL droplets of KSOM-0.03% BSA and treated (37 °C, 5% CO_2_, 30 min) with 2 µM or 6 µM SC-SGG or 6 µM PG. Eggs were then washed successively in droplets of fresh KSOM-0.03% BSA to remove excess SC-SGG or PG and they were added to untreated capacitated sperm (500 µL) in the four-well culture dish. Co-incubation of the gametes and subsequent assessment of fertilization was as described in the first set of experiment. Eggs untreated with either SC-SGG or PG and processed in parallel to those treated eggs served as controls.

In an alternate experiment, the morphology of eggs treated with SC-SGG or PG, as well as untreated eggs, was microscopically assessed as a function of treatment times (1 h, 2 h and 6 h).

#### 4.3.9. Statistical Analyses

Significant differences in data among samples were analyzed by one-way ANOVA with Tukey’s multiple comparison.

## 5. Conclusions

The chemically synthesized SC-SGG was aqueous soluble and exerted inhibitory effects on mouse IVF. These inhibitory effects of SC-SGG on both sperm and eggs and the results corroborated our previous demonstration that sperm SGG is integral to sperm–egg interactions in both the sperm–ZP and sperm–egg plasma-binding steps. SC-SGG has the potential to be developed into a non-hormonal contraceptive.

## Data Availability

All data from this study are contained within this article and Supplemental Information.

## Supplementary Materials

The following supporting information can be downloaded at: https://www.mdpi.com/article/10.3390/ph18050611/s1, Supplementary Video S1. Lack of effects of SC-SGG and PG on motility of capacitated Percoll gradient-centrifuged sperm. Capacitated Percoll gradient-centrifuged (PGC) sperm resuspended in KRB-0.3% BSA at 10 million sperm/mL were treated with 0 and 6 µM SC-SGG or 6 µM PG (37 °C, 5% CO_2_, 10 min). PGC sperm treated with 0 µM SC-SGG served as controls. Approximately, 10 µL of the sperm suspension was pipetted onto a slide and their motility was video-recorded under an inverted microscope using a 10× objective with a Celestron HD Digital Microscope Imager (Torrance, CA, USA) containing a 30× magnifier lens attached to the eyepiece. The video recording for each sample was from 12 to 20 s. Supplementary Material Part 1 contains ^1^H-NMR, ^13^C-NMR and MS spectra of compounds **3**, **4** and **1**. Supplementary Material Part 2 contains the synthetic scheme for the preparation of SGG (1-*O*-palmityl-2-*O*-palmitoyl-3-*O*-[3-*O*-oxysulfonyl-β-d-galactopyranosyl]-*sn*-glycerol. Supplementary Material Part 3 contains the logD graphs of SGG molecular species with various acyl chain length.
