# Supplementary material for: A Short-Chain Analogue of Seminolipid: Synthesis and Inhibitory Effect on Mouse Fertilization
Source: Pharmaceuticals (Basel). 2025 Apr 23;18(5):611. doi: 10.3390/ph18050611 (PMC12114865; doi:10.3390/ph18050611)
Supplement: Supplementary file 1 [file pharmaceuticals-18-00611-s001.zip › pharmaceuticals-3463188-supp/Supplementary Material_FINAL re-submission.pdf]

Supplementary Material

A short-chain analogue of seminolipid: synthesis and inhibitory effect on mouse fertilization

Seung Gee Lee[1], Leila Vahdati[2], Laura Morelli[2], Luigi Panza[3], Federica Compostella[2,†,*] Nongnuj Tanphaichitr[1,4,†,*]

[1] Inflammation and Chronic Disease Program, Ottawa Hospital Research Institute, Ottawa, ON K1H8L6, Canada; selee@ohri.ca (S.L.)

[2] Department of Medical Biotechnology and Translational Medicine, University of Milan, Via Saldini 50, 20133 Milano, Italy; vahdati@gmail.com (L.V.), laura.morelli@unimi.it (L.M.)

[3] Dipartimento di Scienze del Farmaco, Università degli Studi del Piemonte Orientale A. Avogadro, L.go Donegani 2/3, 28100 Novara, Italy; luigi.panza@uniupo.it (LP)

[4] Department of Obstetrics & Gynecology, Faculty of Medicine, University of Ottawa, Ottawa, ON K1H8L6, and Department of Biochemistry, Microbiology, Immunology, Faculty of Medicine, University of Ottawa, Ottawa, ON K1H8M5, Canada

*Correspondence: ntanphaichitr@ohri.ca; Tel: +1 (613) 737-8899 (ext 72793); federica.compostella@unimi.it; Tel: +39-0250316045

† Sharing senior authorship

Content:

Supplementary Material Part 1:

^{1}H-NMR, ^{13}C-NMR, and MS spectra of compounds **3**, **4**, and **1** — page 2-7

Supplementary Material Part 2:

synthetic scheme for the preparation of **SGG** — page 8

^{1}H-NMR, ^{13}C-NMR, and MS spectra of **SGG** — page 9-10

Supplementary Material Part 3:

Calculated logP and logD values of SGG molecular species with various acyl chain length — page 11-12

Supplementary Material Part 1

Compound 3

^{1}H NMR (500 MHz, $CDCl_3$)

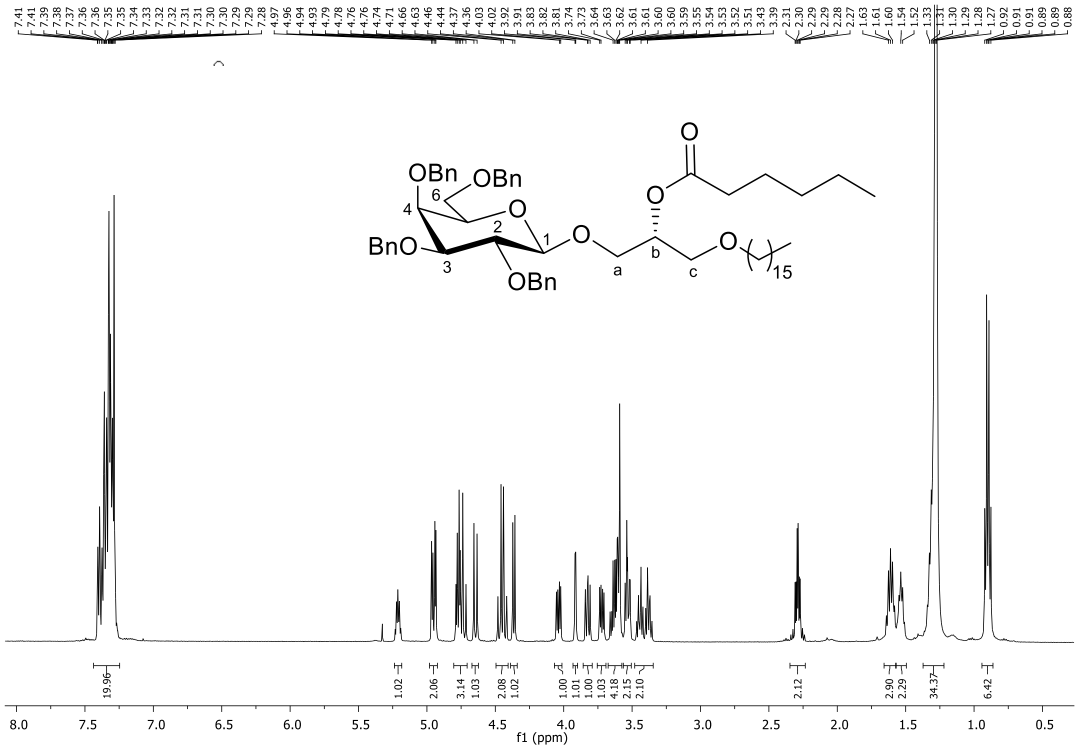

^{13}C NMR (125 MHz, $CDCl_3$)

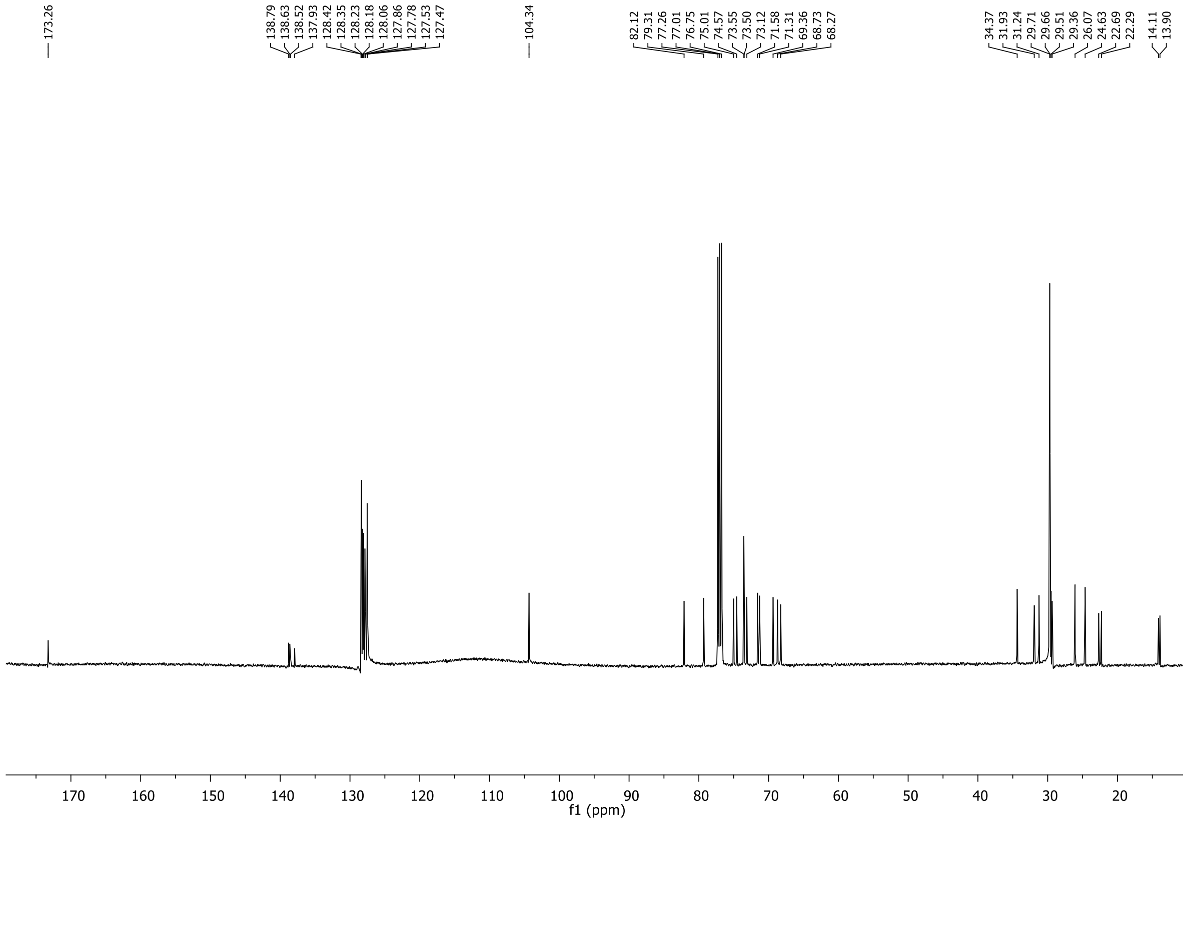

Compound 3

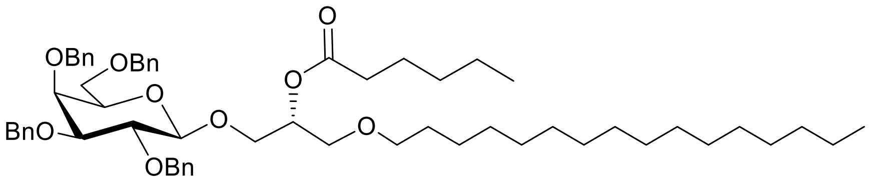

Chemical Formula: $C_{59}H_{84}O_9$
Exact Mass: 936,612
Molecular Weight: 937,312

ESI-MS (positive ion mode)

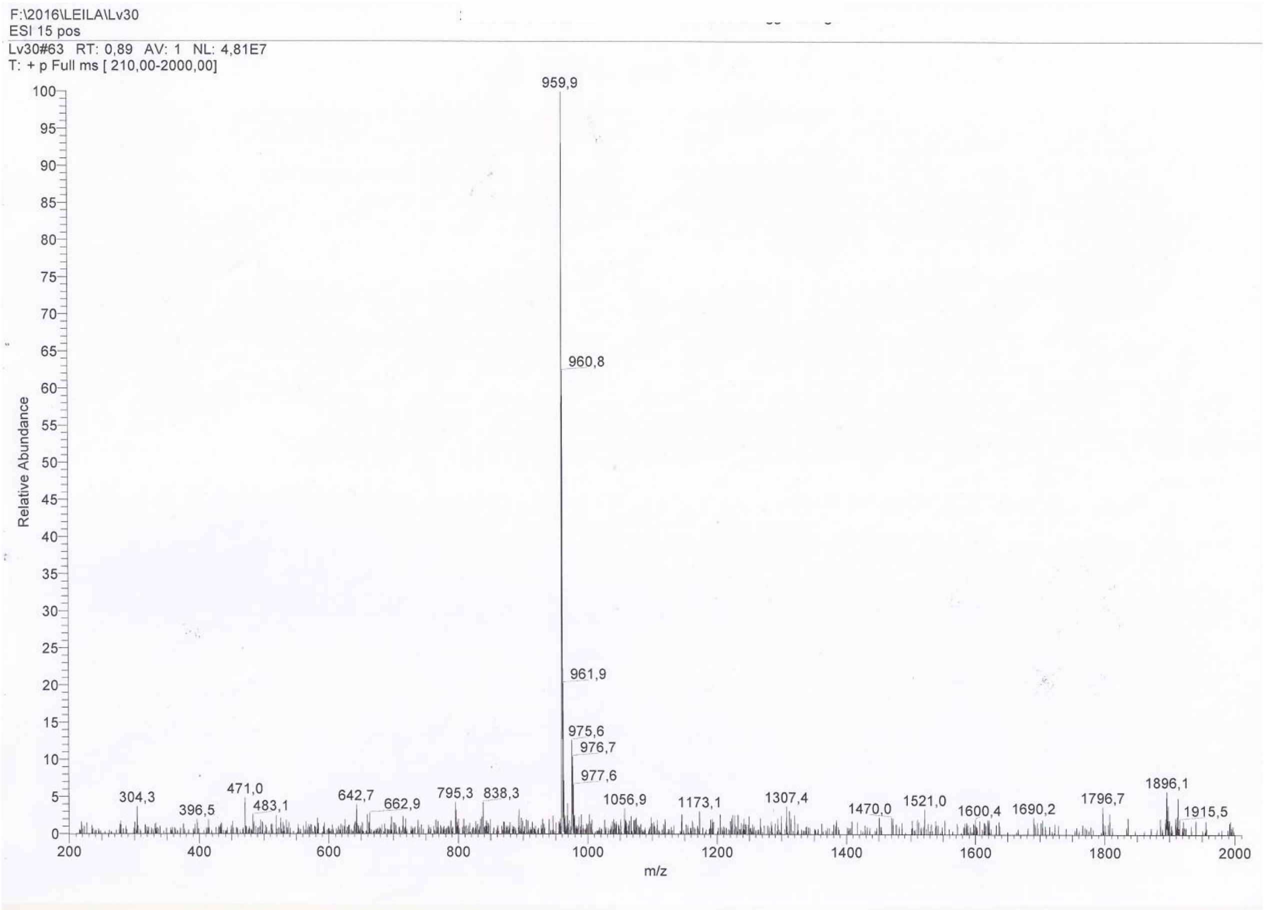

Compound 4

^{1}H NMR (500 MHz, $CDCl_3$)

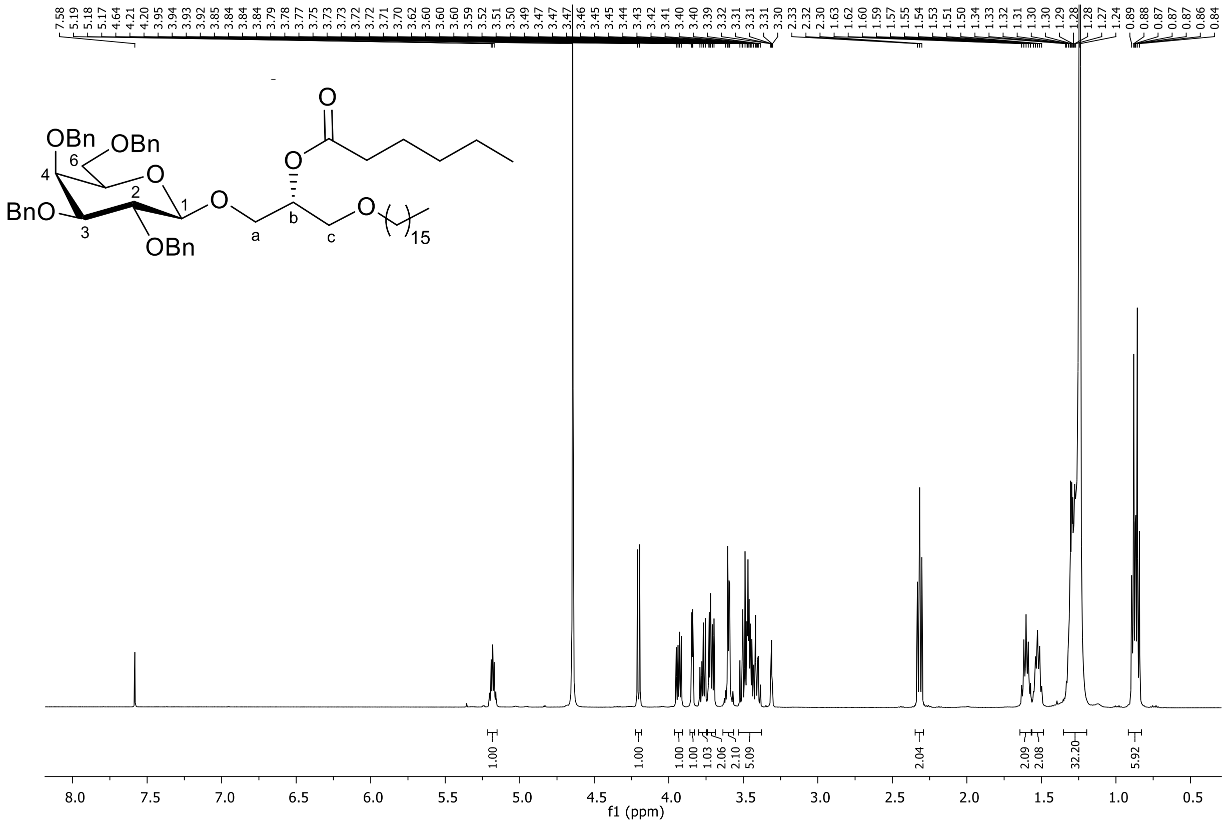

^{13}C NMR (125 MHz, $CDCl_3$)

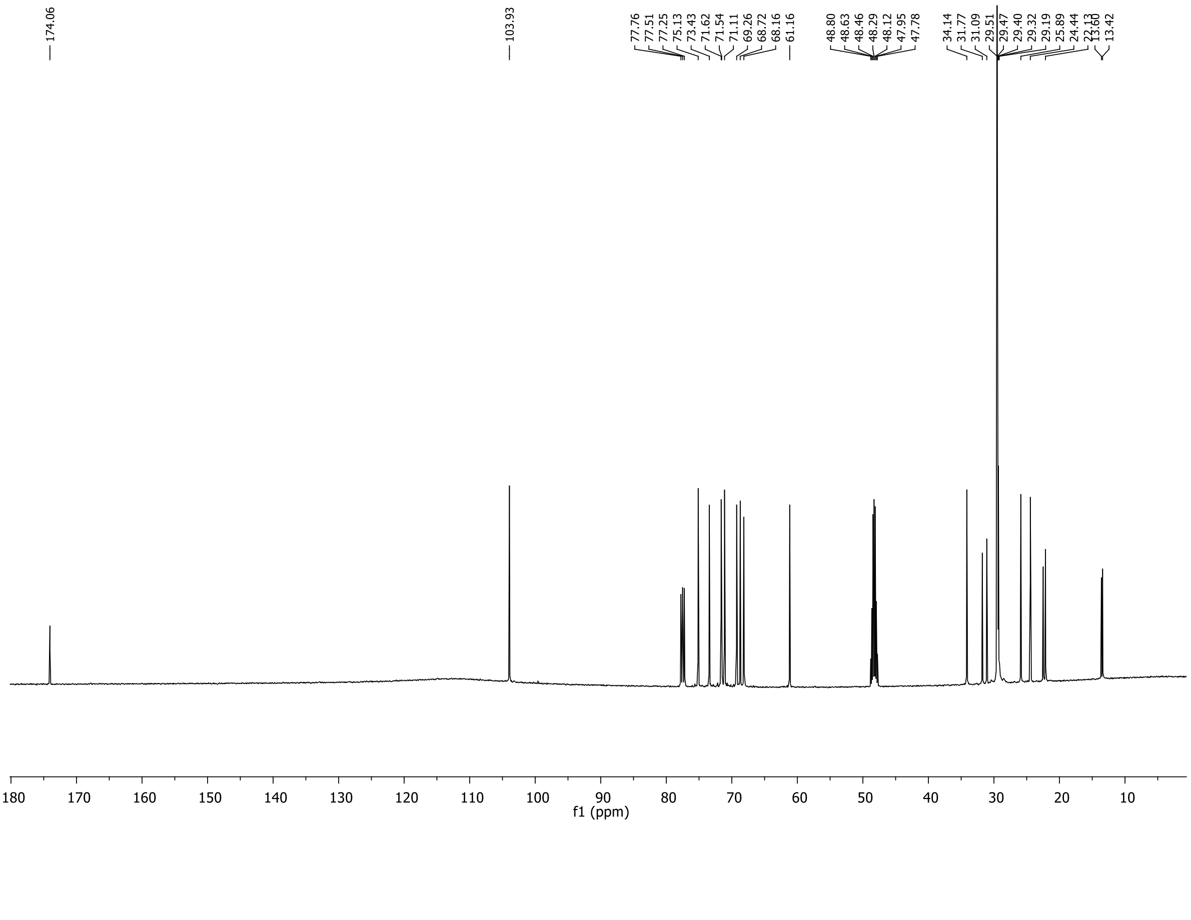

Compound 4

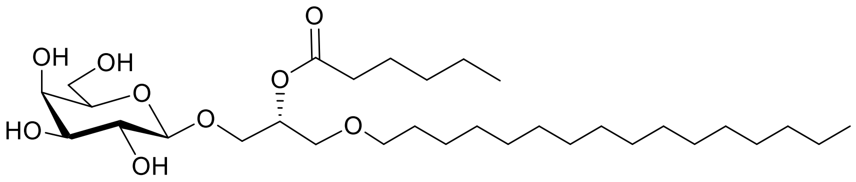

Chemical Formula: $C_{31}H_{60}O_9$
Exact Mass: 576,424
Molecular Weight: 576,812

ESI-MS (positive ion mode)

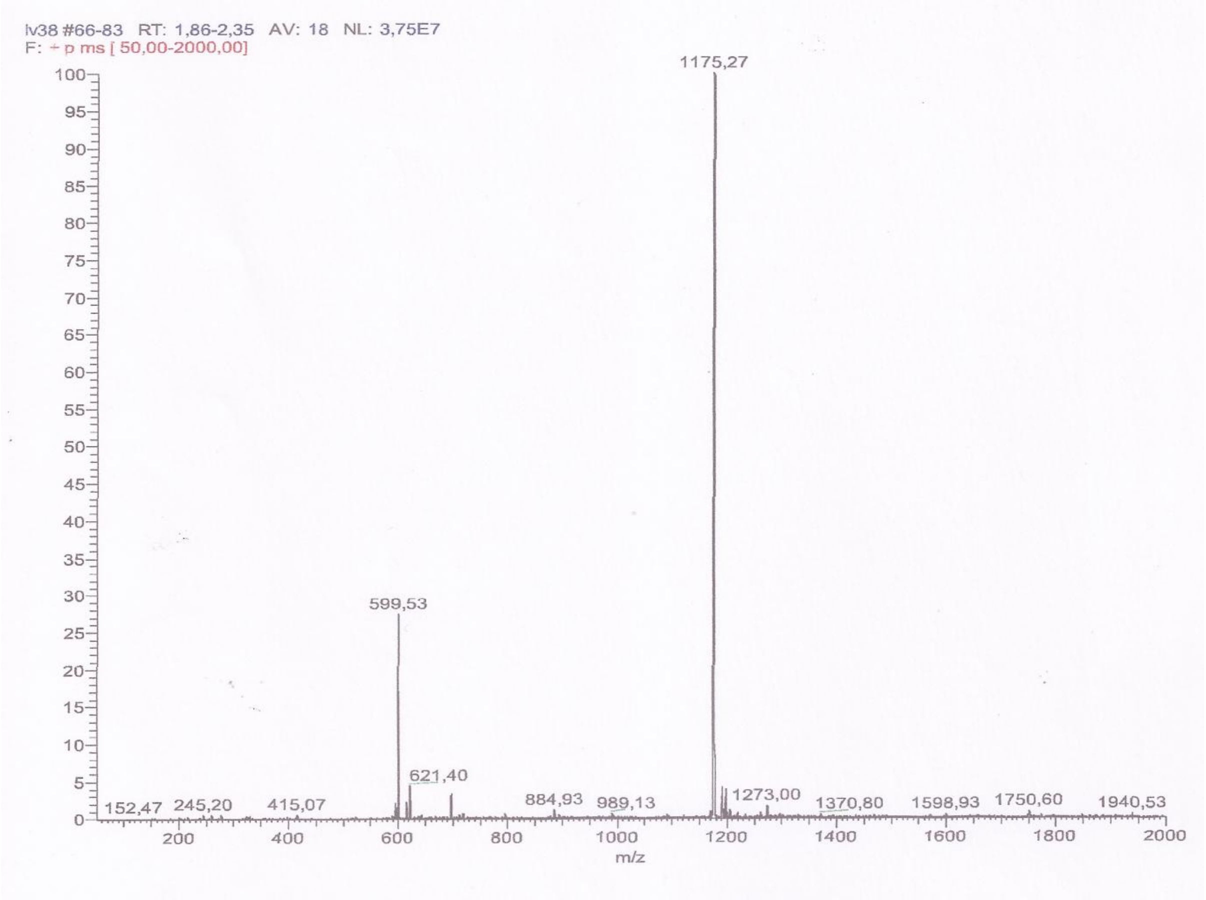

Compound 1 (SC-SGG)

^{1}H NMR (500 MHz, $CDCl_3/CD_3OD$)

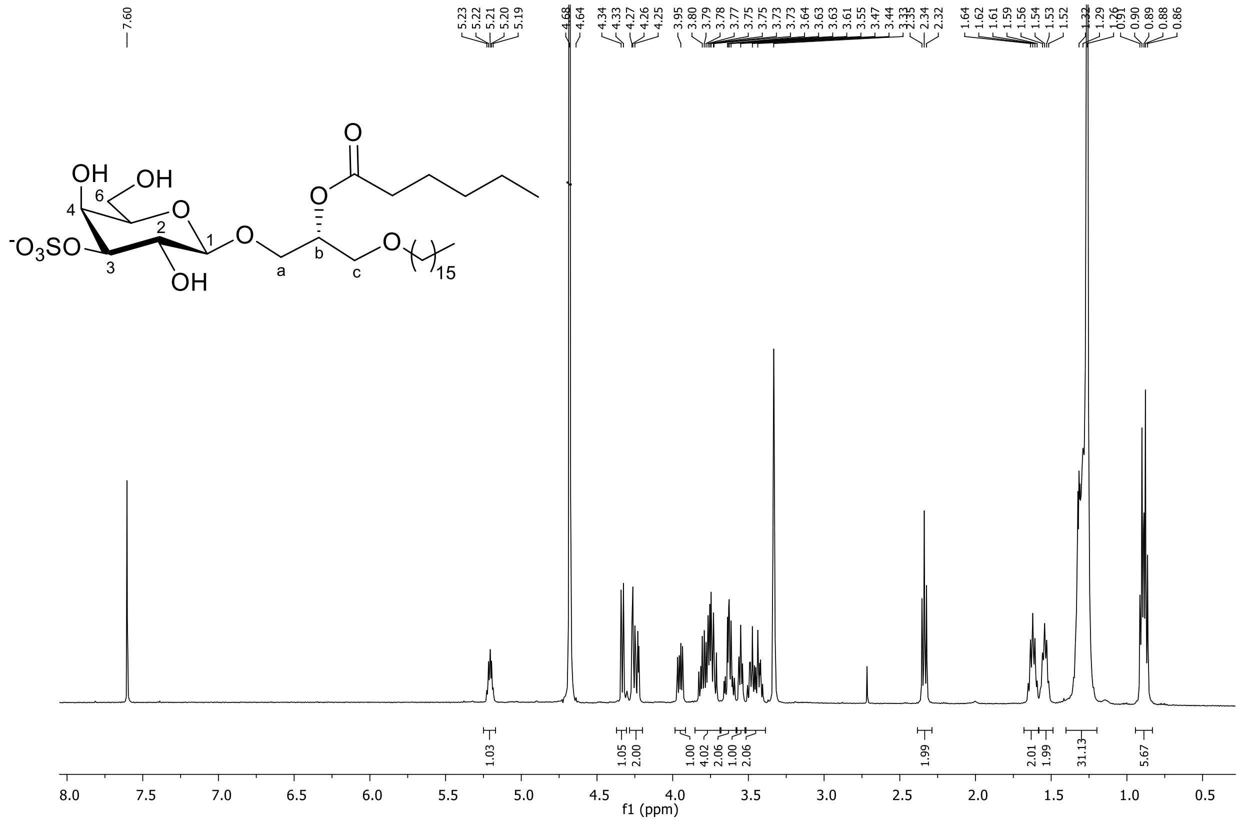

^{13}C NMR (125 MHz, $CDCl_3/CD_3OD$)

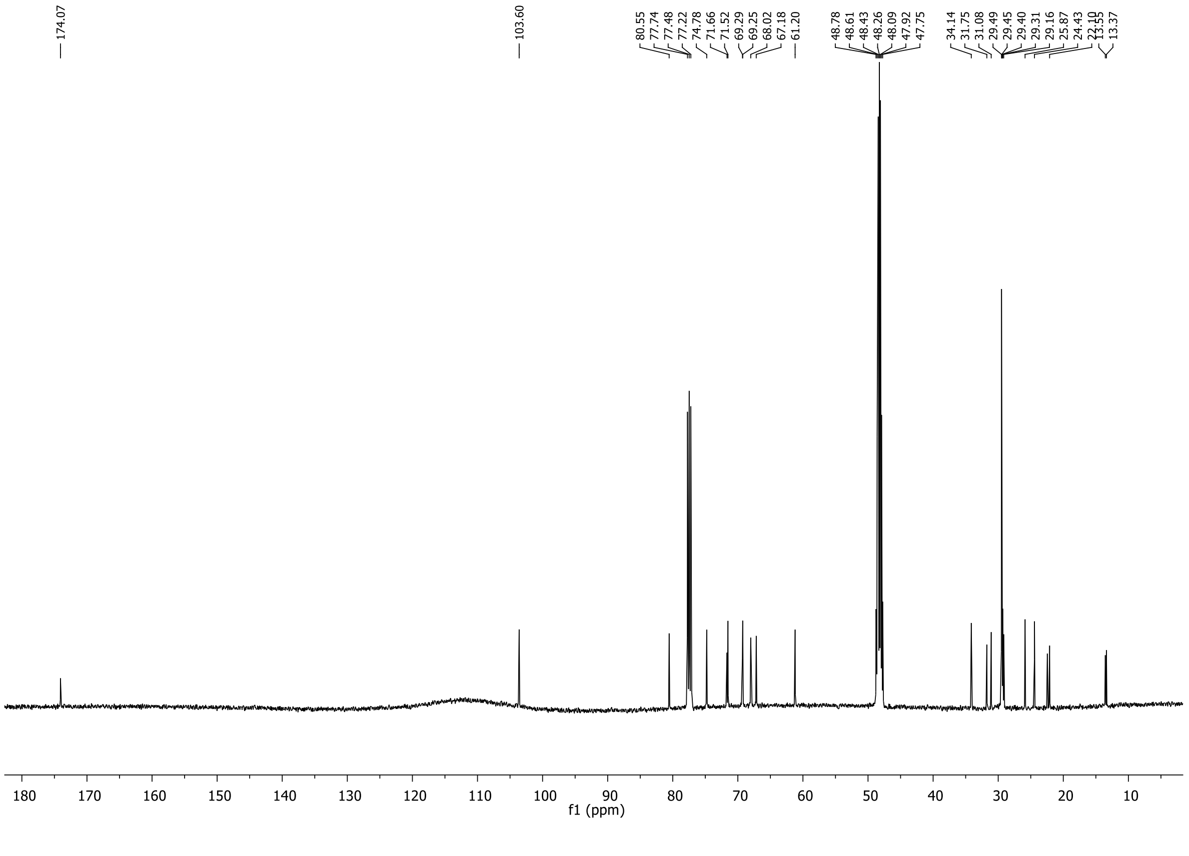

Compound 1 (SC-SGG)

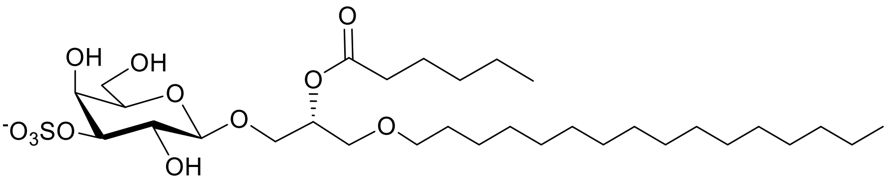

Chemical Formula: $C_{31}H_{59}O_{12}S^-$
Exact Mass: 655,373
Molecular Weight: 655,862

ESI-MS (negative ion mode)

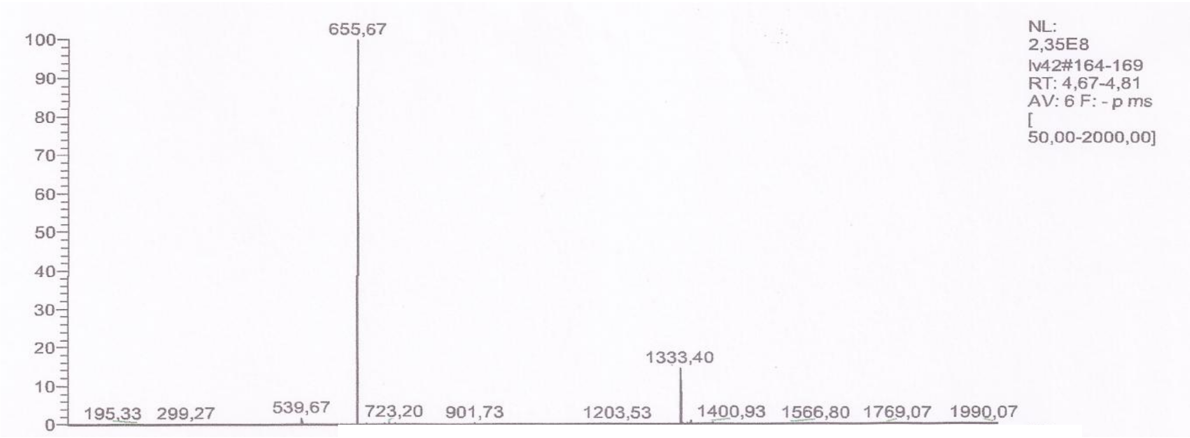

Supplementary Material Part 2

Synthesis of SGG

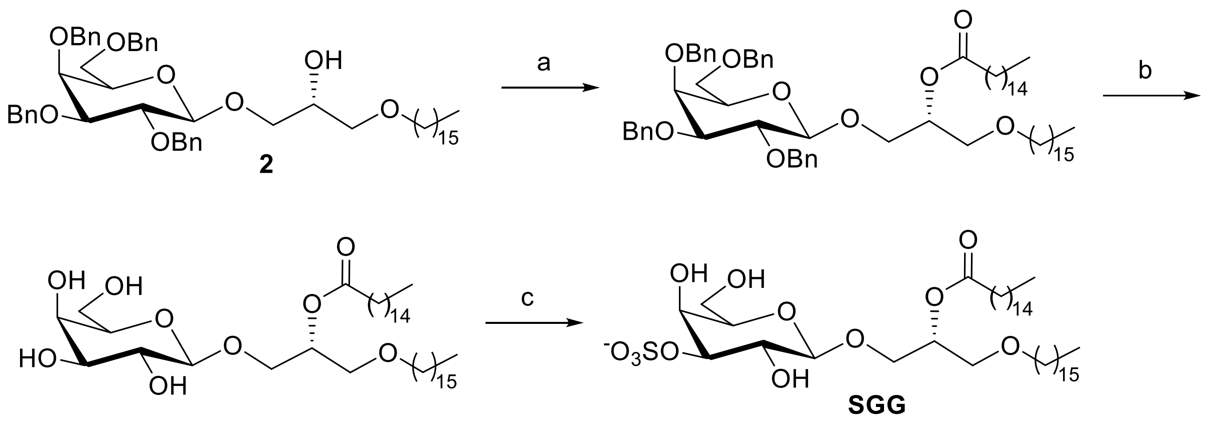

Reagents and conditions: a) Palmitic acid, EDCI, DMAP, CH_2Cl_2, reflux, 83%; b) H_2, Pd/C, MeOH/EtOAc, 95%; c) Bu_2SnO, MeOH, then $Me_3N{\cdot}SO_3$, THF, 83%.

SGG

^{1}H NMR (500 MHz, $CDCl_3/CD_3OD$)

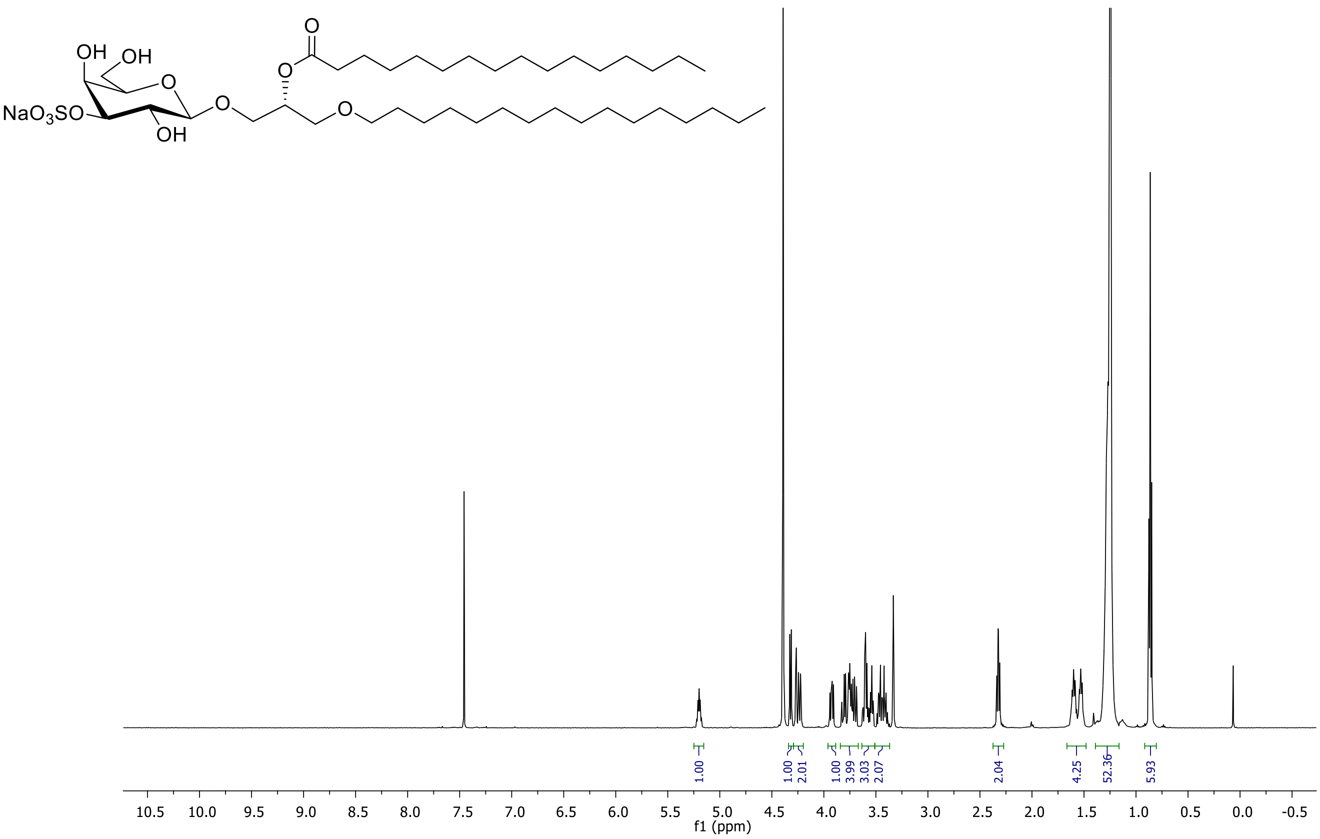

^{13}C NMR (500 MHz, $CDCl_3/CD_3OD$)

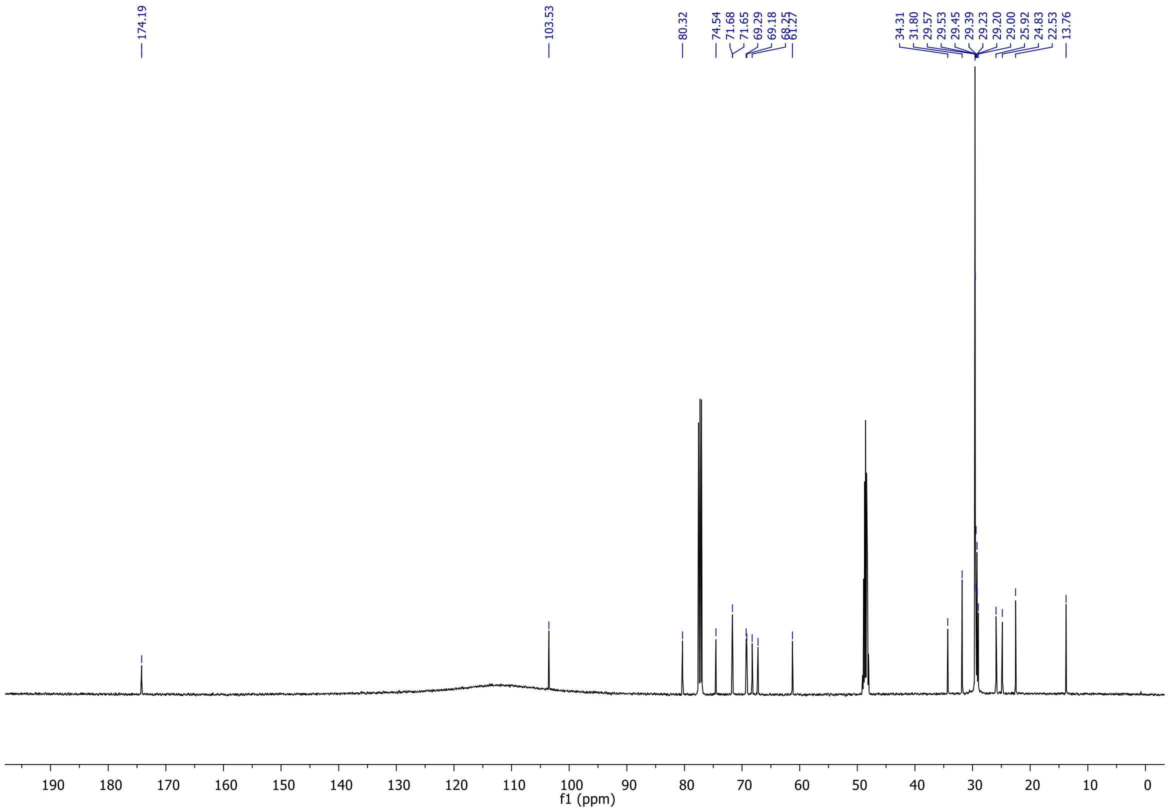

SGG

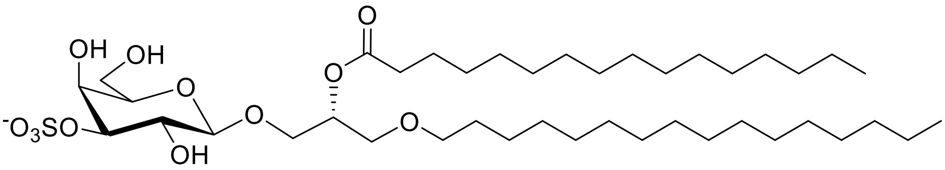

Chemical Formula: $C_{41}H_{79}O_{12}S^-$
Exact Mass: 795,53
Molecular Weight: 796,13

ESI-MS (negative ion mode)

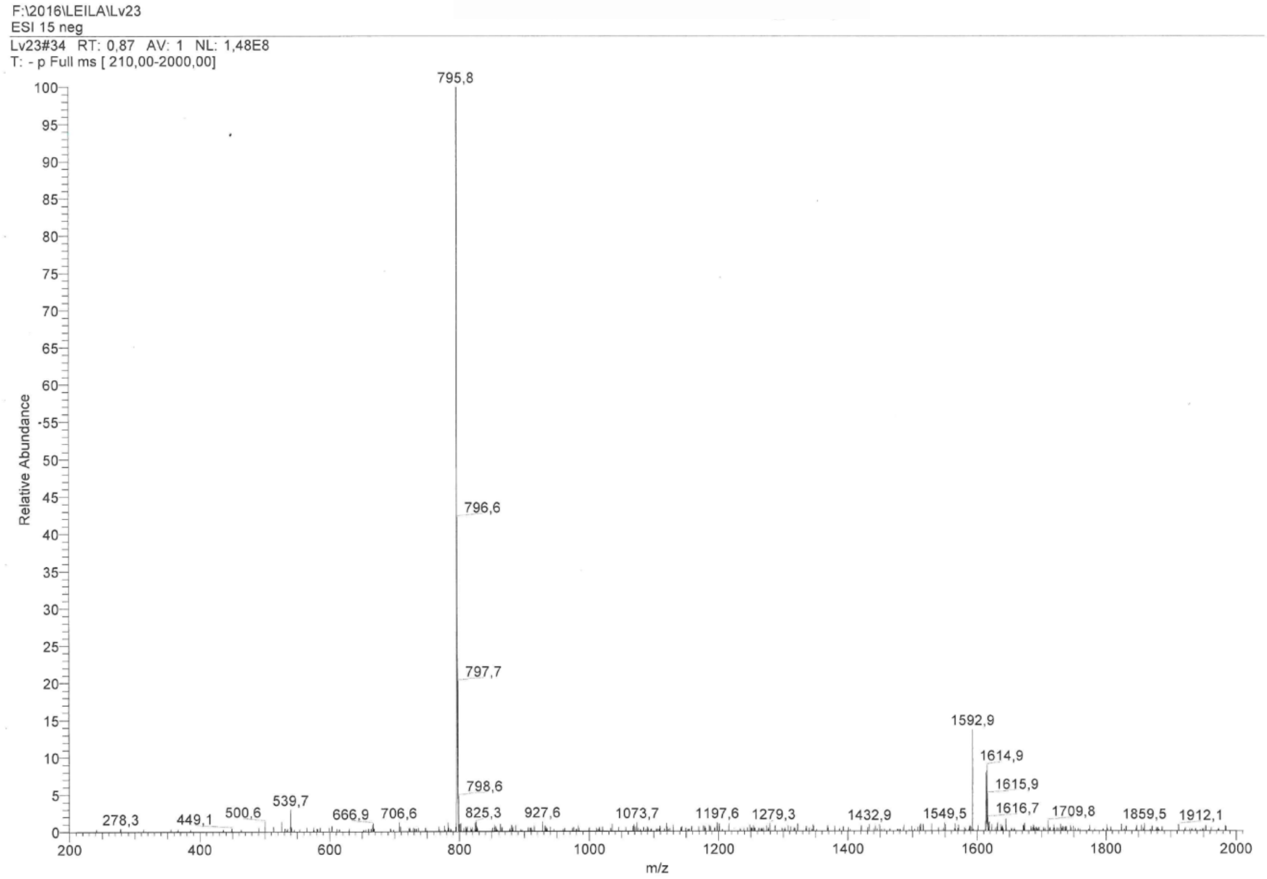

Supplementary Material Part 3

Calculated logP and logD values of SGG molecular species with various acyl chain length

We have theoretically calculated logP and logD values for the set of compounds reported in the Figure:

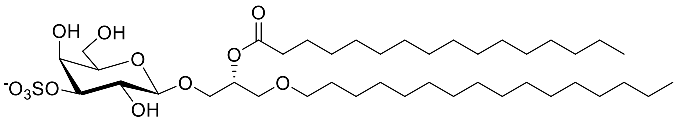

C16-SGG (natural)

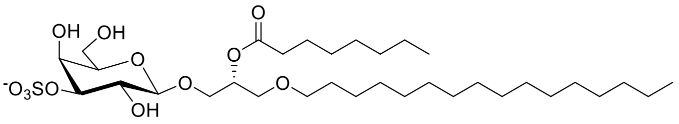

C8-SGG

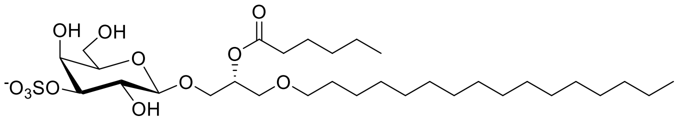

C6-SGG (SC-SGG)

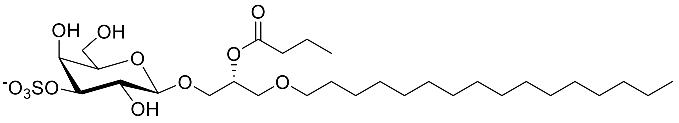

C4-SGG

- LogP values were calculated with the QikPro module from the MAESTRO suite of Schrodinger (version 2023-4).

	logP
C16-SGG (natural)	8.38
C8-SGG	5.38
C6-SGG	3.93
C4-SGG	3.79

- LogD values were calculated with the LogP/LogD calculation suite of the ChemAxon modeling package.

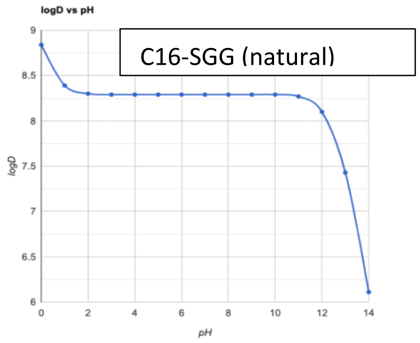

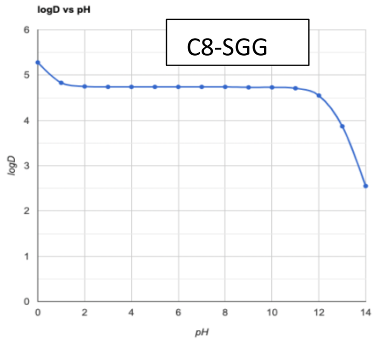

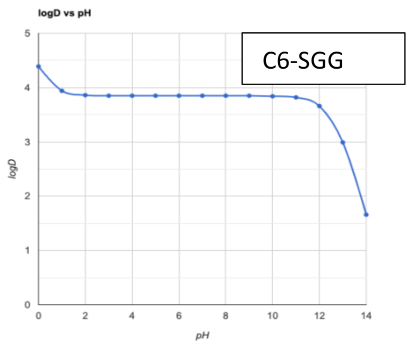
logD vs pH
C6-SGG
logD
pH

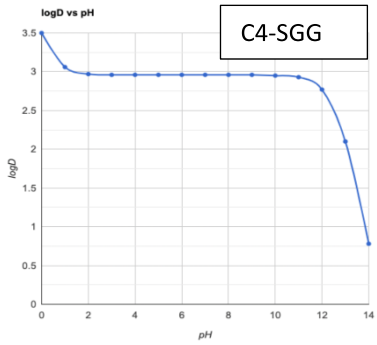
logD vs pH
C4-SGG
logD
pH
