# Supplementary material for: A Short-Chain Analogue of Seminolipid: Synthesis and Inhibitory Effect on Mouse Fertilization
Source: Pharmaceuticals (Basel). 2025 Apr 23;18(5):611. doi: 10.3390/ph18050611 (PMC12114865; doi:10.3390/ph18050611)
Supplement: Supplementary file 1 [file pharmaceuticals-18-00611-s001.zip › pharmaceuticals-3463188-supp/supplementaryvideo1legend.pdf]

Supplementary video 1. Lack of effects of SC-SGG and PG on motility of capacitated Percoll gradient centrifuged sperm. Capacitated Percoll gradient centrifuged (PGC) sperm resuspended in KRB-0.3% BSA at 10 million sperm/mL were treated with 0 and 6 µM SC-SGG or 6 µM PG (37°C, 5% CO_2, 10 min). PGC sperm treated with 0 µM SC-SGG served as controls. Approximately, 10 µl of the sperm suspension was pipetted onto a slide and their motility was videorecorded under an inverted microscope using a 10X objective with a Celestron HD Digital Microscope Imager (Torrance, CA, USA) containing a 30X magnifier lens attached to the eyepiece. The video recording for each sample was from 12 to 20 seconds.
